# Insect collecting bias in Arizona with a preliminary checklist of the beetles from the Sand Tank Mountains

**DOI:** 10.3897/BDJ.11.e101960

**Published:** 2023-06-28

**Authors:** M. Andrew Johnston, Evan S. Waite, Ethan R Wright, Brian H. Reily, Gilma Juanita De Leon, Angela Iran Esquivel, Jacob Kerwin, Maria Salazar, Emiliano Sarmiento, Tommy Thiatmaja, Sangmi Lee, Kelsey Yule, Nico Franz

**Affiliations:** 1 Biodiversity Knowledge Integration Center, Arizona State University, Tempe, AZ, United States of America Biodiversity Knowledge Integration Center, Arizona State University Tempe, AZ United States of America

**Keywords:** Madrean Sky Islands, beetles, natural history museums, biogeography, Sonoran Desert

## Abstract

**Background:**

The State of Arizona in the south-western United States supports a high diversity of insects. Digitised occurrence records, especially from preserved specimens in natural history collections, are an important and growing resource to understand biodiversity and biogeography. Underlying bias in how insects are collected and what that means for interpreting patterns of insect diversity is largely untested. To explore the effects of insect collecting bias in Arizona, the State was regionalised into specific areas. First, the entire State was divided into broad biogeographic areas by ecoregion. Second, the 81 tallest mountain ranges were mapped on to the State. The distribution of digitised records across these areas were then examined.

A case study of surveying the beetles (Insecta, Coleoptera) of the Sand Tank Mountains is presented. The Sand Tanks are a low-elevation range in the Lower Colorado River Basin subregion of the Sonoran Desert from which a single beetle record was published before this study.

**New information:**

The number of occurrence records and collecting events are very unevenly distributed throughout Arizona and do not strongly correlate with the geographic size of areas. Species richness is estimated for regions in Arizona using rarefaction and extrapolation. Digitised records from the disproportionately highly collected areas in Arizona represent at best 70% the total insect diversity within them. We report a total of 141 species of Coleoptera from the Sand Tank Mountains, based on 914 digitised voucher specimens. These specimens add important new records for taxa that were previously unavailable in digitised data and highlight important biogeographic ranges.

Possible underlying mechanisms causing bias are discussed and recommendations are made for future targeted collecting of under-sampled regions. Insect species diversity is apparently at best 70% documented for the State of Arizona with many thousands of species not yet recorded. The Chiricahua Mountains are the most densely sampled region of Arizona and likely contain at least 2,000 species not yet vouchered in online data. Preliminary estimates for species richness of Arizona are at least 21,000 and likely much higher. Limitations to analyses are discussed which highlight the strong need for more insect occurrence data.

## Introduction

Insects represent over half of all described species (Mayhew 2007) and perhaps not more than 20% of those that exist have thus far been described (Gaston 1991). The State of Arizona, located in south-western United States along the Mexico border, has high insect diversity and ranks as the State with the most species actively monitored for conservation (Bossart and Carlton 2002). Entomologists from around the country and around the world travel to southern Arizona every year during the monsoon season (late summer and early fall) where popular canyons may have five to ten campsites and road pull-offs occupied by blacklights and collectors scrambling around them until early morning. Despite its insect diversity and popularity as a collecting destination, we are unaware of any empirical studies that assessed total insect species richness within the State or its subregions or explored biases in insect collecting therein.

Biodiversity occurrence records represent an enormously important, invaluable and irreplaceable data source for understanding biodiversity, evolution and ecology (Cook et al. 2014, Page et al. 2015, Guralnick et al. 2016, Johnston et al. 2018, Kharouba et al. 2018, Meineke et al. 2018, Lendemer et al. 2019, Hedrick et al. 2020). The vast majority of these records, at least for insects, presently come from digitised preserved specimens from natural history collections. However, we know that the specimens stored within collections are not evenly distributed throughout space and time and have many implicit biases intertwined with the history and methods used to accumulate them (Hortal et al. 2015, Johnston et al. 2018, Kharouba et al. 2018, Cooper et al. 2019, Whitaker and Kimmig 2020, Laney et al. 2021, Davis et al. 2022). Human observations have been rapidly increasing thanks to popular platforms such as iNaturalist which have their own slightly different biases, limitations and strengths. We broadly consider fine-scale documentation of individual insects to be "collecting" for the purposes of this paper, though most of our recommendations are focused on traditional preserved-specimen-based collections.

The goals of this study are twofold. First, we present an analysis of digitised insect occurrence data from the State of Arizona and compare the relative levels of sampling for different mountain ranges and ecoregions. Second, we address one example of an underexplored region and provide the first checklist of beetle species from the Sand Tank Mountains of central Arizona. We hope that these data and analyses can inform and bolster future insect collecting to improve our understanding of Arizona's biodiversity.

### Arizona regionalisation

Arizona encompasses a wide array of habitat types ranging from extreme deserts to mesic conifer forests. To efficiently classify these regions, different levels of the hierarchical ecoregions defined by Omernik and Griffith (2014) can be used. Level 3 of those ecoregions gives a broad look at the State and is helpful to consider distributions and collecting efforts in broad strokes (Fig. 1a). However, this level of classification does not account for the fine scale habitat and plant community shifts that are seen, especially in the mountainous parts of the State (see Brown (1978), Brown et al. (2007)).

Arizona can also be regionalised by its many mountain ranges. The Madrean Sky Islands are a series of discrete mountain ranges that arise from surrounding grasslands and are variously forested at their higher elevations (Fig. 1a area 12.1.1). These mountains are situated in the only gap of the North American Cordillera between the Rocky Mountain range to the north and the Sierra Madre Occidental range to the south and are a priority in insect conservation and phylogeographic research (Stock and Gress 2006, Ober et al. 2011, Moore et al. 2013, Halbritter et al. 2019, Yanahan and Moore 2019). Beyond the Madrean Sky Islands, the western and southern parts of Arizona are part of the Basin and Range Province of western North America which is characterised by a large number of mountain ranges that have formed as the Earth's crust stretched in this region (Morrison 1991) and which covers the Sonoran and Mojave Deserts (Fig. 1a areas 10.2.1 and 10.2.2). A final series of mountains occur in Arizona along the Mogollon Highlands region (Fleischner et al. 2017) which is a slightly oblique area of plateaus and associated mountains that generally separates north-eastern Colorado Plateau from the southern Basin and Range Province (Fig. 1a area 13.1.1 in centre of the State).

Outlines of the 81 mountain ranges in Arizona with the highest peaks were geographically mapped for use in this study and are shown in Fig. 1b. The shapefiles of Arizona ecoregions and mountain ranges now allow for exploration of digitised insect occurrence records (Fig. 1c, d) to understand underlying patterns in bias and diversity of these areas.

### Sand Tank Mountains

The Sand Tank Mountains, located in south central Arizona (Fig. 1b label 78, Fig. 2), cover a moderately large area in the Lower Colorado River Basin region of the Sonoran Desert (Brown 1978). The mountain range is situated with roughly its northern half on the Sonoran Desert National Monument bounded by US Interstate 8 to the north and its southern half on the Barry M. Goldwater Air Force Range. The mountains are, therefore, nearly entirely on public land, though access and collecting largely requires permits from the latter two entities. The highest point in the range, Maricopa Peak, only reaches 1234 m in elevation. The mountains are named for a series of tanks or tinajas (natual stone water catchments) that were often largely filled with sand and typically available to wildlife and humans for most of the year (Bryan 1925: 224-228).

Very little is known about the fauna of the Sand Tank Mountains and the adjoining Sauceda Mountains to the southwest (Fig. 1b, label 75) which are almost never mentioned in the scientific literature. Brown (1978) included the Sand Tank mountains in a list of lower-elevation Sonoran Desert ranges which had relictual patches of grassland and chapparal species on them. The Sand Tanks also are the location of a notable Jaguar (*Pantheraonca* (Linnaeus, 1758), family Felidae) record from 1930 which represents the south-western known limit of the species in the State and likely the furthest documented excursion of the species into the Sonoran Desert (Babb et al. 2022).

Prior to the study presented here, a total of 27 occurrence records representing 16 insect taxa were available online (GBIF 2022a). This includes only a single record for the order Coleoptera which represents nearly 25% of all described species on Earth, from a photo voucher on iNaturalist. We were unable to find any other beetle records from the mountains in the published scientific literature or in our own work in Arizona natural history collections.

## Materials and methods

### Data sources and region delimitation

Occurrence records for insects (Fig. 1) were downloaded from the online aggregator Global Biodiversity Information Facility (GBIF). Records were downloaded from GBIF (GBIF 2022a) by searching for every record that had geographic coordinates, contained 'Arizona' in the stateProvince data field and that belonged to Class Insecta, resulting in a dataset of 712,309 occurrence records. GBIF was chosen as the only data source for this analysis in part because of its versioned DOI for downloads and also because it provided the most records of any other portal. The Integraded Digitised Biocolections (iDigBio) portal contains 612,142 records using the same search parameters and the Symbiota Collections of Arthropods (SCAN) portal contains 683,645 records, nearly all of which are overlapping between the portals. The GBIF mediated data are further enhanced by their backbone taxonomy which is a synthetic management classification for the portal (GBIF Secretariat 2022). All records are harmonised to the GBIF taxonomy which helps to clean misspellings and differently formatted data from the various data contributors making diversity and species richness estimates more plausible. However, the influence of the GBIF taxonomy is influential in another way since there are so many taxonomic names that are not yet known to GBIF. This may affect as many as 75% of records and names for major insect orders (Waller 2022).

The occurrence records were imported into qGIS 3.24 (qGIS Development Team 2022) and checked against shape files with polygons representing ecoregions from the United States EPA (Omernik and Griffith 2014) and mountain ranges within Arizona. The list of mountain ranges was generated primarily by consulting online resources for mountain climbers. A curated list of mountain ranges and their highpoints (Anonymous 2022) was used as the starting point and each range was verified through a combination of United States Geological Survey (USGS) topographic maps, Google Maps searches and consulting regional gazetteers and atlases. Our working definition of a mountain range for the purposes of biological regionalisation is as follows: a geographically contiguous string of mountains which seem to have a shared geological origin and are separated from other such groups by a lower elevational region which appears to have different geology and/or vegetative cover as assessed via satellite imagery. These mountain ranges typically matched very closely those labelled on topographic maps and gazetteers. Polygons for each mountain range were drawn by hand around geological formations as viewed in satellite imagery; topographic maps from the USGS, personal experience in the field and mountain range and place names in google maps were used to ascertain a polygon that represented the footprint around the mountain range. Shifts in geology and vegetative cover were especially helpful to define the periphery of mountain ranges. Our definitions attempted to delimit potentially biologically meaningful entities more than they were an attempt to perfectly outline the underlying geology. Any occurrence found within the footprint of one of the included shapes was annotated as such. A custom script (Suppl. material 5) was written in R 4.0.2 (R Core Team 2020) to summarise the number of records by area metrics. Figures were produced in the same R script and utilised the packages dplyr (Wickham et al. 2022), ggplot2 (Wickham 2016) and cowplot (Wilke 2020).

### Evaluating digitised records for collecting bias

For entomological field work, differences in occurrence records likely reveal a compilation of biological differences (e.g. increased insect biomass and population densities would increase the number of occurrence records), differences in survey effort (e.g. one area may have been visited by 100 researchers a year and another area by 10 researchers per year) and differences in social practices and research interests (e.g. one person may collect 100 of 200 observed individual insects at a particular event, while another person may collect 5 of 200 observed individual insects at a different event). Insect occurrence records were, therefore, analysed according to three different metrics, namely records, collecting events and species. First, the total number of occurrence records for a given ecoregion or mountain range were tallied as a sum. Second, collecting effort was approximated by pooling records into putative collecting events. All insect records from a particular ecoregion or mountain range that had an identical date (using dwc:day, month and year fields) and collector (dwc:recordedBy field) were considered to belong to a single collecting event. Third, putatively unique insect taxa were totalled for each ecoregion and mountain range by counting unique scientific names (dwc:scientificName field). These names correspond to the taxonomic interpretation according to the GBIF backbone taxonomy. This count may be considered an overestimate because different individuals of the same taxon may have been identified to different ranks (e.g. subspecies, species, genus and family) and be counted multiple times. However, because so many taxa at the species level are not known to the GBIF taxonomy, many differently identified taxa are prone to being 'lumped' into a higher classification level (Waller 2022). For studies where the goal is to create a verified checklist of names, the original verbatim data from individual providers are included on GBIF, but we deemed the taxon names as filtered by GBIF to be more standardised and at least easily comparable across ecoregions and mountain ranges. All data are made available as supplemental materials for annotated occurrence records (Suppl. material 1), summarised data for ecoregions (Suppl. material 2) and mountain ranges (Suppl. material 3).

Sampling effort to geographic area relationships were explored using linear regressions of both total occurrence records and tabulated collecting events to geographic area of regions (both for ecoregions and mountain ranges). A linear fit with high correlation would indicate that insect collecting was evenly distributed throughout space, while stronger departures from such a relationship would indicate confounding factors affecting the distribution of insect sampling across the State. There is some debate about what the most appropriate models are for species-area relationships and growing evidence suggests that very small, intermediate and very large areas operate under very different scaling parameters (Lomolino 2000, Lomolino and Weiser 2001, Stiles and Scheiner 2007, Dengler 2009, Storch et al. 2012, Storch 2016, Dengler et al. 2019). Different scaling parameters could warrant analysing data under different transformations. Our study is primarily focused on understanding the scale and bias of insect records as they relate to geographic areas in Arizona and, therefore, presents somewhat simplistic explorations of the data as a first step towards future studies which may employ more complex models to explore specific biological questions. However, we did assess our dataset for normality since different analytical techniques might apply to these data depending on the underlying biological power laws at play (García Martín and Goldenfeld 2006, Packard 2014). The untransformed data were not normal, but the log-transformed data were. Normality assessments and analyses on log-transformed data and plots of species by geographic area of EcoRegions and mountain ranges are available in (Suppl. material 6).

Possible factors responsible for underlying bias within the occurrence records were assessed using the R package sampbias (Zizka et al. 2020) to examine how spatial distribution of roads, cities, airports and rivers might affect where insects are collected. The analyses were run using all georeferenced insect records for the State using default settings within sampbias which performs a Bayesian analysis to determine the range of posterior probabilities for how each factor biases the underlying dataset. The bias each factor introduces is then compiled into a spatial model for an expected sampling effort given the calculated biases. The resulting bias model was calculated for Arizona and was then visualised along with a heatmap of insect occurrence records for the State.

Species richness within areas was estimated using the R package iNEXT (Chao et al. 2014, Hsieh et al. 2020) to perform species rarefaction and extrapolation analyses. Counts were tabulated for the total number of records for each unique taxon within a region and these abundance data were given to iNEXT and analysed using q = 0 for the appropriate Hill number estimation for abundance data (Chao et al. 2014). Our analyses were primarily focused on exploring relative completeness of species richness sampling found within occurrence data, but future studies primarily interested in modelling precise species richness would likely need to explore records in more detail to discern where there is and is not overlap at different taxonomic scales (e.g. how should records to the genus level be counted if a single species from that genus is already counted from the area?). We analysed taxa as unique name strings as described above for all analyses. We further re-analysed several areas with a more conservative approach where we only used the subset of records that were identified to species (i.e. dwc:taxonRank = SPECIES) to explore how that changed extrapolation of total species richness. None of the rarefaction and extrapolation analyses presented here approaches an asymptote within an estimated doubling of sampling effort and, therefore, has limitations in truly accounting for unobserved taxa in species richness estimates (Willis 2019); nevertheless, the rarefaction curves and estimates are still useful tools to understand uses and limitations of the underlying data.

### Checklist of Sand Tank Mountains Coleoptera

Three collecting trips were made to the Sand Tank Mountains to survey for beetles. The first was on 29 April 2022 where blacklighting and night searches with headlamps were performed in a rocky basin near a paved wildlife water catchment basin (32.7868, -112.5177). Uncovered pitfall traps were set here and in a wide sandy wash (32.7982, -112.5112, Fig. 2d). The second trip was a single overnight visit from 3-4 June 2022 where the previously deployed pitfall traps were collected and night collecting was performed in the sandy wash site involving beating vegetation, blacklighting and night searching. The third and final trip was on 15 July 2022 to a rocky canyon in which lies Bender Spring (32.6786, -112.3657, Fig. 2c) where blacklighting and night searches were performed. All beetle specimens were mounted and labelled and then identified to the lowest level possible. Full data for every voucher used in this study are fully digitised in the Ecdysis portal built on the Symbiota software (Gries et al. 2014), published to GBIF (GBIF 2022b) and in Suppl. material 4. Most specimens were deposited in the Arizona State University Hasbrouck Insect Collection (ASUHIC) with duplicates and focal research taxa deposited in the M. Andrew Johnston Research Collection (MAJC), Evan Waite Invertebrate Collection (EWIC) and Ethan Richard Wright Collection (ERWC), all of which are located in Tempe, Arizona, USA. Identifications were typically made using Arnett et al. (2002) to the level of family and genera. Species-level identifications were then performed by using appropriate primary literature or by consulting local taxonomic experts. The final identification resource for each species in the checklist is provided. For taxa identified by experts where a specific source is unknown, we attribute the identification to that person as unpublished data.

The checklist of species was built using the Ecdysis portal checklist tool from all of the digitised specimen records created as part of this project. The curated checklist was then exported for publication and inserted into the ARPHA writing platform for this journal. Families are presented in alphabetical order and all species are presented alphabetically under their family. A total of 140 new species level records were identified, anchored by 914 fully digitised pinned and labelled voucher specimens. When combined with the previously available record, the following checklist enumerates 141 species of Coleoptera from the mountain range.

## Checklists

### Sand Tank Mountains Coleoptera

#### Anthicidae

4A8AD8C0-EF9F-553A-9C7A-50D17D153E8E

#### Duboisius arizonensis

(Champion, 1916)

556E25A5-4CC2-5842-B2D8-25EA91D5084B

##### Notes

Identification reference: Abdullah (1964)

#### Duboisius barri

Abdulluh, 1964

9DE05AE5-8AC0-532D-BFA4-273D28B930EA

##### Notes

Identification reference: Abdullah (1964)

#### Notoxus calcaratus

Horn, 1884

ADEBC8E0-FD06-5ACC-939F-0EBFFE742546

##### Notes

Identification reference: Chandler (1982)

#### Vacusus confinis

(LeConte, 1851)

ED0ACD7E-3616-5B56-BD4A-62C2413FAE0D

##### Notes

Identification reference: Werner (1961)

#### Bostrichidae

44EE7654-4228-5276-99EA-296FC2DDCFDD

#### Amphicerus cornutus

(Pallas, 1772)

D5BB4323-283D-50E5-A43C-F56B9A0AE9DB

##### Notes

Identification reference: Fisher (1950)

#### Amphicerus teres

Horn, 1878

59153EBE-F94F-5C62-A02F-12C9B7C98254

##### Notes

Identification reference: Fisher (1950)

#### Apatides fortis

(LeConte, 1866)

72BD7E41-A4A6-58CC-99E3-F512873F3346

##### Notes

Identification reference: Arnett et al. (2002)

#### Dendrobiella aspera

(LeConte, 1858)

1D8D04B9-919D-5075-9B4A-686C4CA9A001

##### Notes

Identification reference: Fisher (1950)

#### Xyloblaptus quadrispinosus

(LeConte, 1866)

74D18D55-634F-5FF4-BA35-08BCBF7DCCC5

##### Notes

Identification reference: Fisher (1950)

#### Brachypsectridae

4D1D0564-0000-5FAE-B184-2B75F488687D

#### Brachypsectra fulva

LeConte, 1874

94ADD790-C17C-5338-8CB6-B41A29EDFA4C

##### Notes

Identification reference: Costa et al. (2006)

#### Buprestidae

543D471A-7219-5B1D-8197-57B89736E24E

#### Chrysobothris micromorpha

Fall, 1907

3F0D8D9D-FFF3-585F-9992-DEBEF358210F

##### Notes

Identification reference: Arnett et al. (2002), N. Woodley personal communication to MAJ 2022.

#### Gyascutus caelatus

(LeConte, 1858)

41295C3C-5841-595F-8BDE-14E7DFE688B4

##### Notes

Identification reference: iNaturalist

#### Melanophila atropurpurea

(Say, 1823)

DC32ADD5-F707-5A46-B432-73F30E9F3E45

##### Notes

Identification reference: Sloop (1937)

#### Carabidae

C0590844-1327-59B3-943E-8828636BF14F

#### Apristus sp.

12B1BF5D-B133-5685-A8F2-629D2EF9222F

##### Notes

This genus is in need of revision and we were unable to identify our specimens beyond genus.

#### Bembidion impotens

Casey, 1918

D982D779-39A6-5F75-9167-A0707BDBE1EC

##### Notes

Identification reference: Lindroth (1963)

#### Calosoma prominens

LeConte, 1853

92B3741D-EA08-5146-8EFA-9D3F2EFD1A39

##### Notes

Identification reference: Gidaspow (1959)

#### Chlaenius orbus

Horn, 1871

1468E917-6936-53F4-884D-0346499AFC5D

##### Notes

Identification reference: Bell (1960)

#### Cymindis punctigera

LeConte, 1851

A1612B58-1D3C-556F-99B7-8231F0ED4232

##### Notes

Identification reference: Horn (1882)

#### Discoderus obsidianus

Casey, 1914

5E5A1AC8-B1F7-58D3-B69F-6D9F8D14DF24

##### Notes

Identification reference: Casey (1914)

#### Elaphropus conjugens

(Notmann, 1919)

144769EF-D9E3-5942-8E6F-F0BBE0FD0E06

##### Notes

Identification reference: Erwin (1974)

#### Lebia tuckeri

(Casey, 1920)

D6A636DE-4F78-50C8-82FA-C9B9D0120B5E

##### Notes

Identification reference: Madge (1967)

#### Notiobia terminata

(Say, 1823)

DB2496C0-2E7B-58F5-961B-C4B23AA1D0C7

##### Notes

Identification reference: Noonan (1973)

#### Schizogenius pygmaeus

Van Dyke, 1925

9A61B35C-74B9-5D4B-814E-30B914071F1C

##### Notes

Identification reference: Whitehead (1972)

#### Selenophorus aeneopiceus

Casey, 1884

96B0087C-7F02-5C20-87CC-8C29D4EA92A1

##### Notes

Identification reference: Messer and Raber (2021)

#### Selenophorus concinnus

Schaeffer, 1910

A9FD2932-D3E0-53C5-B4A6-CB95D81F4E81

##### Notes

Identification reference: Messer and Raber (2021)

#### Tetragonoderus pallidus

Horn, 1868

6675B9A6-1DCA-5838-917D-C5DFCFE03CFE

##### Notes

Identification reference: Horn (1881)

#### Cerambycidae

D2928EA4-E2BA-52D1-93A8-048B5572DA14

#### Anelaphus albofasciatus

(Linell, 1897)

404DC5A1-DBDE-5DDB-91C8-CE62FD95F49A

##### Notes

Identification reference: F.W. Skillman unpublished data.

#### Anelaphus brevidens

(Schaeffer, 1908)

2EC2C75A-9A92-58F0-9842-8B25B4E38168

##### Notes

Identification reference: F.W. Skillman unpublished data.

#### Anelaphus piceus

(Chemsak, 1962)

5CD51E8C-788C-5582-B17F-02B8CF3B01E3

##### Notes

Identification reference: F.W. Skillman unpublished data.

#### Anelaphus submoestus

Linsley, 1942

63BAF14E-32AB-5396-BFBD-CA02FD8322F8

##### Notes

Identification reference: F.W. Skillman unpublished data.

#### Anoplocurius canotiae

Fisher, 1920

FD9D01F7-9B68-52AD-B9C3-86430C11E428

##### Notes

Identification reference: F.W. Skillman unpublished data.

#### Eustromula validum

(LeConte, 1858)

B6D95084-AF19-5BD9-B7AB-B772E6DE9A49

##### Notes

Identification reference: F.W. Skillman unpublished data.

#### Methia brevis

Fall, 1929

F4FAD1C4-4CD1-5EAD-9AF8-BE8794E5B280

##### Notes

Identification reference: F.W. Skillman unpublished data.

#### Moneilema gigas

(LeConte, 1873)

CB2F6A6C-267B-5D9A-BBA4-10882537F753

##### Notes

Identification reference: F.W. Skillman unpublished data.

#### Sternidius centralis

(LeConte, 1884)

75627271-F5EF-5D7F-82D7-374F0A537F5D

##### Notes

Identification reference: F.W. Skillman unpublished data.

#### Chrysomelidae

FE15B802-4C44-5816-82D0-A848D899B9F5

#### Colaspis viridiceps

Schaeffer, 1933

64A8BF95-1A06-5281-A9A2-7F1174384144

##### Notes

Identification reference: Blake (1976)

#### Coleorozena sp.

68C32E26-AB47-54C1-BC50-6D70DF036AB0

##### Notes

This genus needs revision and we were unable to identify our specimens beyond genus.

#### Coleothorpa axillaris

(LeConte, 1868)

9564A5AC-A180-5A3B-8A8C-E2161C641A6B

##### Notes

Identification reference: Moldenke (1970)

#### Diorhabda carinulata

(Desbrochers, 1870)

084AA5D4-4C37-5422-B2AC-CFC4745B5695

##### Notes

Identification reference: TRACY and ROBBINS (2009)

#### Glyptina sp.

ED88B8C9-384B-5385-AEBA-4D43C2ACD3DA

##### Notes

This genus needs revision and we were unable to identify our specimens beyond genus.

#### Pachybrachis connexus

Fall, 1915

923E5AB1-0EEB-5E42-AEF5-D0B39D40BAA9

##### Notes

Identification reference: Barney (2019)

#### Pachybrachis mellitus

Bowditch, 1909

1C36C4C7-F759-5838-B629-786C16E0FE8F

##### Notes

Identification reference: Fall (1915)

#### Pachybrachis vigilans

Fall, 1915

F71EC8E7-CCE7-584B-AE83-59EB9EE19CFC

##### Notes

Identification reference: Barney (2019)

#### Pachybrachis wickhami

Bowditch, 1909

52002F17-5CAF-5669-9B48-DF132BAF7B2C

##### Notes

Identification reference: Barney (2019)

#### Pachybrachis xanti

Crotch, 1873

FD29851E-63CF-54ED-B246-3D2056B7E924

##### Notes

Identification reference: Barney (2019)

#### Saxinis saucia

LeConte, 1857

BB59A861-5348-583F-8940-F3BD4F13B4B7

##### Notes

Identification reference: Moldenke (1970)

#### Cleridae

BA894C10-BDE8-5DC5-8A0B-35C4C497C525

#### Araeodontia peninsularis

(Schaeffer, 1904)

E476C649-DCCC-5BDC-9A8B-757BA7CB732D

##### Notes

Identification reference: B.H. Reily unpublished data.

#### Cymatodera latefascia

Schaeffer, 1904

BDC25CC1-656F-52BC-B8BD-01AE02E10B9B

##### Notes

Identification reference: B.H. Reily unpublished data.

#### Cymatodera oblita

Horn, 1876

8A1BE50C-D93F-58FB-82A0-A32F45DE8A8C

##### Notes

Identification reference: B.H. Reily unpublished data.

#### Cymatodera punctata

Leconte, 1852

E00F2696-3D98-5A16-9958-213D33E1E496

##### Notes

Identification reference: B.H. Reily unpublished data.

#### Enoclerus quadrisignatus

(Say, 1835)

1DE00017-E9EE-5A22-B2F0-5ED12C45794B

##### Notes

Identification reference: B.H. Reily unpublished data.

#### Lecontella gnara

Wolcott, 1927

B8924B82-45EF-5898-ADAC-A9FD60F1E0CB

##### Notes

Identification reference: B.H. Reily unpublished data.

#### Phyllobaenus discoideus

(LeConte, 1852)

CFE93839-BB5E-504F-BDD5-27768617EB20

##### Notes

Identification reference: B.H. Reily unpublished data.

#### Coccinellidae

FF55D192-DA90-5F8F-B3CC-8F3DFF8F43D0

#### Hyperaspis pleuralis

Casey, 1899

0248D2CE-31F3-50CD-B46F-6C0C9F6C9A1C

##### Notes

Identification reference: Gordon (1985)

#### Scymnus sp.

16D20865-5784-5F97-993D-F50F9265BFFF

##### Notes

Identification of this genus requires examination of male terminalia. Our single putatively female specimen was only identified to the subgenus Scymnus (Pullus), of which there are a number of species known from this region.

#### Curculionidae

F9F98C8D-0787-58BD-B0C2-81A880FEBC89

#### Eucyllus unicolor

Van Dyke, 1936

1FEF5503-BA54-50E3-B531-2B97AF1F7F2D

##### Notes

Identification reference: Pelsue and Sleeper (1972)

#### Ophryastes sp.

C6F9AB97-6A4F-581A-AC1E-BC365BB12828

##### Notes

This diverse genus is difficult to identify without genitalic dissections and we were unabe to identify our specimen to species.

#### Rhinostomus frontalis

(LeConte, 1874)

5A16EF68-9108-549D-AEAE-DBEE8E6CE87B

##### Notes

Identification reference: Morrone and Cuevas (2002)

#### Sibinia simplex

(Casey, 1892: 421)

A45D0113-EBDF-56E4-925B-5DFE05895964

##### Notes

Identification reference: Clark (1978)

#### Sibinia transversa

(Casey, 1897: 665)

659D2D8E-B677-544A-AD7E-14B66887F80E

##### Notes

Identification reference: Clark (1978)

#### Smicronyx sp.

075D76CA-278D-5C51-B062-62A2031D8C3E

##### Notes

This speciose genus is difficult to identify and we were unable to identify our single specimen beyond genus.

#### Dascillidae

DBE5116C-4EF8-59B5-AF04-99A67F441892

#### Anorus parvicollis

Horn, 1894

9B7B87BE-6067-5DEE-9BA5-33E3749F27BE

##### Notes

Identification reference: Johnston and Gimmel (2020)

#### Dermestidae

06BB1F55-5478-5100-89F3-8400BD41E36C

#### Anthrenus lepidus

LeConte, 1854

746DEC1C-94E7-5972-9535-447486950F20

##### Notes

Identification reference: Beal (1998)

#### Dytiscidae

41388122-8EB8-5F0D-A13F-93E267686A05

#### Eretes sticticus

(Linnaeus, 1767)

57833888-4F01-5023-8EF9-FF61BD0DC2B5

##### Notes

Identification reference: Miller (2002)

#### Elateridae

A8D46359-8E4D-5070-810A-B4383F62DEE3

#### Agrypnus illimis

(Horn, 1894)

DB3DD9A7-ECE8-540E-BD30-D3C728D49D64

##### Notes

Identification reference: Arnett (1952)

#### Anchastus bicolor

LeConte, 1866

51382046-AAD4-535D-B70F-75ED0D7D6326

##### Notes

Identification reference: Van Dyke (1932)

#### Esthesopus parcus

Horn, 1884

E014B0D5-DED6-5184-ABF4-E5365FD133E6

##### Notes

Identification reference: Horn (1884)

#### Horistonotus lutzi

Van Dyke, 1933

70B3D25E-09CF-53E7-BD53-45B7ABB371FB

##### Notes

Identification reference: Wells (2000)

#### Horistonotus simplex

LeConte, 1863

35B52708-FE26-5B32-A082-44E8A8653F29

##### Notes

Identification reference: Wells (2000)

#### Mulsanteus arizonensis

(Schaeffer, 1916)

7F8F22D6-54AD-5643-A912-CDB9208A2F17

##### Notes

Identification reference: B. Mathison unpublished data.

#### Histeridae

672FCEB9-E0E6-5FFB-ABC7-83F09801D5BB

#### Xerosaprinus coerulescens

(LeConte, 1851)

26D8F485-4A6D-5940-B20A-D5986D61F022

##### Notes

Identification reference: W.B. Warner unpublished data.

#### Xerosaprinus martini

(Fall, 1917)

E59B4134-D477-5102-ADC6-4F3DC2B64B2C

##### Notes

Identification reference: W.B. Warner unpublished data.

#### Meloidae

F494A362-59ED-56B6-BB6E-25513AC71401

#### Epicauta lauta

(Horn, 1885)

3BCC05C8-3458-5AEA-B1E6-5D6434A643A6

##### Notes

Identification reference: Werner et al. (1966)

#### Epicauta tenebrosa

Werner, 1949

03759E26-447F-5239-8295-0B5BC36F8EFD

##### Notes

Identification reference: Werner et al. (1966)

#### Epicauta tenuilineata

(Horn, 1894)

4B845E05-5AF1-500A-A7D9-F0CA4F757E4F

##### Notes

Identification reference: Werner et al. (1966)

#### Epicauta virgulata

(LeConte, 1866)

2994E3C5-B23F-5B0D-AA67-7EFE4F5719B9

##### Notes

Identification reference: Werner et al. (1966)

#### Pyrota trochanterica

Horn, 1894

B2D571A7-36D4-52EB-A7A2-F41AEE4A79EE

##### Notes

Identification reference: Werner et al. (1966)

#### Melyridae

7986BF5F-7F50-52F3-900D-8259B4C4436D

#### Attalus serraticornis

Fall, 1917

4CEF76F9-F368-5BB0-A816-0E6DFA443D24

##### Notes

Identification reference: Marshall (1951)

#### Trichochrous ferrugineus

(Gorham, 1886)

A3809ABC-8830-513D-AE31-A8C1C08FCFD4

##### Notes

Identification reference: M.L. Gimmel unpublished data.

#### Trichochrous varius

Casey, 1895

2D3C39C8-0E0B-50AC-A351-F7AEC2161BE6

##### Notes

Identification reference: M.L. Gimmel unpublished data.

#### Mordellidae

460467DE-C8CD-5A27-AFCF-ACCBF2BB5014

#### Mordellina sp.

9D76D9E6-D421-578A-BBAB-4042DB4DCE84

##### Notes

This genus has limited identification resources available. Our two specimens resemble *Mordellinatestacea* (Blatchley, 1910) - a species only reported from the eastern United States.

#### Mycetophagidae

60732CED-843C-5110-BF65-01DF6C758CA3

#### Typhaea stercorea

(Linnaeus, 1758)

62EECD02-029A-5D4C-B66A-8CFF6D031B81

##### Notes

Identification reference: Arnett et al. (2002)

#### Oedemeridae

98889A98-C8D5-5381-8E14-B87F1D921B1F

#### Oxacis cana

(LeConte, 1866)

C9D22D30-3385-51CF-9A6F-C5E18ACC9F1E

##### Notes

Identification reference: Arnett (1951)

#### Oxacis laevicollis

Horn, 1896

019B0571-13AF-538B-805E-F20F6E125DD0

##### Notes

Identification reference: Arnett (1951)

#### Oxycopis mariae

(Arnett, 1951)

4769596F-149E-5347-9A8F-DCE483853E9D

##### Notes

Identification reference: Arnett (1951)

#### Oxycopis sp.

7F996EDB-A72E-5FA4-B9AA-56D8F62EED8F

##### Notes

A moderate series of this *Oxycopis* species likely represent an undescribed species which we were unable to associate with any currently known from the western United States.

#### Phengodidae

BBA07FCB-13C8-5C07-A0E6-6243F66884EB

#### Distremocephalus opaculus

(Horn, 1895)

3D1F2C5A-A762-50F8-9D44-C9FF78C77B23

##### Notes

Identification reference: Zaragoza Caballero (1986)

#### Ptinidae

7A2C428A-2E5F-5636-922F-14717066F3CD

#### Niptus ventriculus

LeConte, 1859

1827E3D0-B40D-5E85-BB6F-DA74E7373B3A

##### Notes

Identification reference: Aalbu and Andrews (1992)

#### Ptinus paulonotatus

Pic, 1904

F9C85703-5510-56BC-AA04-037EA4318257

##### Notes

Identification reference: Papp (1962)

#### Tricorynus sp.

0049847F-36B7-5DB1-B044-585FFD254E66

##### Notes

This speciose genus is in need of a modern revision. Our single specimen has elytra that lack discernible striae and may be near *Tricorynuslentus* (Fall, 1905).

#### Pyrochroidae

F850DD1B-5D15-547A-94D5-AA86DEE2F780

#### Cononotus bryanti

Van Dyke, 1939

3012C7AC-E344-5E45-BCF7-05407807A0AE

##### Notes

Identification reference: Van Dyke (1939)

#### Salpingidae

D6F87308-AA02-5F49-83E3-26F9B8F1C844

#### Dacoderus striaticeps

LeConte, 1858

D02ACFDD-A798-59C9-A918-56465900A32D

##### Notes

Identification reference: Aalbu et al. (2005)

#### Scarabaeidae

43465072-5D42-5F34-B5D5-1E0024FBE770

#### Acoma arizonica

Brown, 1929

9E3E930E-FE41-519F-BAB3-7173D59B30C5

##### Notes

Identification reference: W.B. Warner unpublished data.

#### Ataenius desertus

Horn, 1871

2606E611-4459-57A1-B56B-47302106810A

##### Notes

Identification reference: W.B. Warner unpublished data.

#### Ataenius hirsutus

Horn, 1871

F1FD620C-BB9B-5011-99CE-7B3DEF27D33B

##### Notes

Identification reference: W.B. Warner unpublished data.

#### Ataenius lobatus

Horn, 1871

ADA0E144-76B2-5E37-A3DA-304FFA1F6984

##### Notes

Identification reference: W.B. Warner unpublished data.

#### Diplotaxis fissilabris

Fall, 1909

E5C6193E-B3A5-5A70-8D76-66D6C90B153C

##### Notes

Identification reference: W.B. Warner unpublished data.

#### Diplotaxis moerens

LeConte, 1856

058EE653-F3BD-5996-A2AF-346375D480B8

##### Notes

Identification reference: W.B. Warner unpublished data.

#### Diplotaxis planidens

Fall, 1909

20728724-F8BF-5049-B123-47D01BAFCD50

##### Notes

Identification reference: W.B. Warner unpublished data.

#### Diplotaxis rufiola

Fall, 1909

4092E1F7-1867-51AA-8814-B1B415CEE252

##### Notes

Identification reference: W.B. Warner unpublished data.

#### Haroldiataenius lucanus

(Horn, 1871)

97CFCF65-B7A0-5946-8637-74E0C7260D70

##### Notes

Identification reference: W.B. Warner unpublished data.

#### Oxygrylius ruginasus

(LeConte, 1856)

AA94F8BE-0DA8-5925-B0A4-5DA1807CA488

##### Notes

Identification reference: W.B. Warner unpublished data.

#### Phyllophaga scoparia

(LeConte, 1856)

D32801FA-FA35-52C2-9E84-00EAE1D62B92

##### Notes

Identification reference: W.B. Warner unpublished data.

#### Scraptiidae

7C0DFA9B-5636-5F55-8A39-1ED3E6E334E1

#### Allopoda sp.

34D22EAB-F896-5736-990E-5C881DB7D8B5

##### Notes

This genus lacks a comprehensive key to species, but genitalic dissections indicate that our series of specimens likely represent an undescribed species.

#### Diclidia greeni

Liljeblad, 1918

16CAF229-1C6F-56EC-8982-105BD06CBC3E

##### Notes

Identification reference: Liljeblad (1945)

#### Naucles pusio

(LeConte, 1858)

46409E3D-4069-556D-8DEC-7CF76B84E86B

##### Notes

Identification reference: Liljeblad (1945)

#### Silvanidae

33AA5FD2-EAA1-541C-A935-0BDF5DEE3700

#### Ahasverus sp.

D4159739-69D6-5494-87DE-9BD54C900656

##### Notes

Our specimens somewhat resemble *Ahasverusrectus* (LeConte, 1854), but differ in several characters from the holotype of that species. We have seen conspecific specimens to ours labelled as "*Ahasverus n.sp.*" in collections and think it is likely that it is, indeed, an undescribed species.

#### Staphylinidae

6C5ABF1D-5C48-511F-8223-49D7BCB5F338

#### Philonthus sp.

30C154DB-2220-50F2-87B8-FDA50CFAEF85

##### Notes

We were unable to identify our single specimen of this species beyond the level of genus in this speciose group.

#### Tenebrionidae

2F91223E-3060-51C1-A034-86E43910BF0F

#### Alaephus macilentus

Casey, 1891

C14DF586-636B-5346-B9C5-5BBD84F55E2C

##### Notes

Identification reference: M.A. Johnston unpublished data.

#### Anepsius delicatulus

LeConte, 1851

5B5C7FB8-0683-5600-8A16-D1BB66CF25B0

##### Notes

Identification reference: Doyen (1987)

#### Araeoschizus decipiens

Horn, 1890

8E907738-172D-5015-97DF-15919066181E

##### Notes

Identification reference: Papp (1981)

#### Araeoschizus regularis

Horn, 1870

C3B93157-CF3E-5FCE-89AF-FB5CABDB22B7

##### Notes

Identification reference: Papp (1981)

#### Argoporis bicolor

(LeConte, 1851)

384F5C44-B87A-54B7-A359-0588EACBAB70

##### Notes

Identification reference: Berry (1980)

#### Asbolus mexicanus angularis

(Horn, 1894)

B2E50530-D8F9-5065-A52B-308959080B5C

##### Notes

Identification reference: Aalbu (2005)

#### Batuliodes rotundicollis

(LeConte, 1851)

1EFE660A-59FD-5833-8480-38FB6052A935

##### Notes

Identification reference: Doyen (1987)

#### Chilometopon helopioides

(Horn, 1870)

5F324B4A-851D-5276-B762-F69889691C48

##### Notes

Identification reference: MacLachlan and Olson (1990)

#### Conibius opacus

LeConte, 1866

1280A39C-60A2-57DA-8A87-CFFD1E69C9A3

##### Notes

Identification reference: M.A. Johnston unpublished data.

#### Craniotus pubescens

LeConte, 1851

7F8F9A08-D6BA-50EF-B94C-D1CA19460E00

##### Notes

Identification reference: Arnett et al. (2002)

#### Cryptoglossa muricata

(LeConte, 1851)

CDDB6901-CD28-532D-BBE7-0D0A98AA6A7D

##### Notes

Identification reference: Aalbu (2005)

#### Cryptoglossa variolosa

(Horn, 1870)

613D250E-BED6-53D7-B1A3-4EF903944C7B

##### Notes

Identification reference: Aalbu (2005)

#### Edrotes ventricosus

LeConte, 1851

AEE14280-EE76-5D35-878D-C3C8BC26C507

##### Notes

Identification reference: Doyen (1968)

#### Eleodes armata

LeConte, 1851

682CC6C1-647A-5F95-ACD8-EC2D6D507510

##### Notes

Identification reference: Johnston et al. (2015)

#### Eleodes tribula

Thomas, 2005

B68460A5-FD34-57E5-B684-7E6277F244F9

##### Notes

Identification reference: Johnston (2016)

#### Eupsophulus castaneus

(Horn, 1870)

149F3F34-C10A-5A53-8C78-00AC3D675775

##### Notes

Identification reference: Spilman (1959)

#### Eurymetopon rufipes

Eschscholtz, 1831

9C41B1C7-DFBA-5498-8F26-010391111D24

##### Notes

Identification reference: M.A. Johnston unpublished data.

#### Eusattus dubius

LeConte, 1851

3D3A992F-3ADB-5C4C-82FC-6D83591EE858

##### Notes

Identification reference: Doyen (1984)

#### Hymenorus papagonis

Fall, 1931

A6FF3988-BFEB-57A1-A761-A5323E65F897

##### Notes

Identification reference: Fall (1931)

#### Hymenorus punctatissimus

LeConte, 1866

71D63690-DBEE-59EA-84DB-335C642CC72A

##### Notes

Identification reference: Fall (1931)

#### Hymenorus spinifer

Horn, 1894

BAA53322-0722-5F1A-AB73-37CB7C65666B

##### Notes

Identification reference: Fall (1931)

#### Latheticus oryzae

Waterhouse, 1880

AFDF615F-7F43-50C1-B94A-8917F38B0D67

##### Notes

Identification reference: Arnett et al. (2002)

#### Nocibiotes caudatus

Casey, 1895

F27E356E-D4B8-5362-9B2F-BE90638AAB5F

##### Notes

Identification reference: M.A. Johnston unpublished data.

#### Nocibiotes granulatus

(LeConte, 1851)

A721C9D2-A5FE-53DB-AE9B-EE8A41033C43

##### Notes

Identification reference: M.A. Johnston unpublished data.

#### Statira defecta

Schaeffer, 1905

CE60184A-9B50-52AA-8425-AE8217617630

##### Notes

Identification reference: Parsons (1975)

#### Statira pluripunctata

Horn, 1888

2CBB4229-416C-5A42-B432-1856447B7856

##### Notes

Identification reference: Parsons (1975)

#### Steriphanus subopacus

(Horn, 1870)

8DAAA19C-9C0F-57C2-BC7C-F6614E9DA41C

##### Notes

Identification reference: M.A. Johnston unpublished data.

#### Telabis longipennis

(Casey, 1890)

A813A5C8-CA37-58DB-8EB4-0812C89DA5C9

##### Notes

Identification reference: M.A. Johnston unpublished data.

#### Triorophus laevis

LeConte, 1851

D1262B1E-EE42-5756-8C7D-AB15107A0CB5

##### Notes

Identification reference: M.A. Johnston unpublished data.

#### Zophobas subnitens

(Horn, 1874)

BFE4EC01-8C02-595A-BB98-67F6E39E5693

##### Notes

Identification reference: M.A. Johnston unpublished data.

#### Trogidae

4E73616A-9186-55DD-9069-EEBC996D0639

#### Omorgus carinatus

Loomis, 1922

D76BA4BB-4887-50E3-9ED0-ED3287DA4F9F

##### Notes

Identification reference: W.B. Warner unpublished data.

#### Trogossitidae

9F2F5549-9F14-5AD0-A1FA-167FDD2BE4A5

#### Temnoscheila chlorodia

(Mannerheim, 1843)

BAB8BD68-4224-5628-9EF4-931BAEE9AE1D

##### Notes

Identification reference: Barron (1971)

#### Zopheridae

94C38543-DFB4-51AC-A451-882022EBAD80

#### Hyporhagus gilensis

Horn, 1872

3C544C6A-BE65-5672-A42F-30DF0F85FB4E

##### Notes

Identification reference: Freude (1955)

#### Rhagodera costata

Horn, 1867

AD592433-0514-5662-B0ED-4145289DD199

##### Notes

Identification reference: Stephan (1989)

## Analysis

### Notes on the Sand Tank Mountains Coleoptera

The checklist provided herein significantly raises the entomological knowledge of this mountain range. Our collecting efforts unfortunately were comprehensively limited as they did not include sampling during the peak flowering season that typically occurs between late February and April or in the winter which has a distinct insect fauna that often does not overlap with the taxa found during the warmer times of year. We also were unable to access a number of distinct habitats, including the relictual chaparral plant communities, that likely would have greatly increased our taxon count.

Many of the species reported from this study occur throughout the Lower Colorado River Basin subregion of the Sonoran Desert, but are often poorly represented in natural history collections or in digitised occurrence records. Six species recorded by us had no prior digitised records from the State of Arizona even though they are known in literature (*Diclidiagreeni*, *Horistonotuslutzi*, *Mulsanteusarizonensis*, *Niptusventriculus*, *Oxycopismariae* and *Ptinuspaulonotatus*). Many more represent the second digitised record or the first preserved specimen, as opposed to a human observation, from the State. These are notable in that they demonstrate specific examples of how digitised records both fall short of representing the full knowledge of the State's fauna, as well as the limited distributional information available for many species. It is also notable that three collecting events produced likely three undescribed species (*Ahasverus* sp., *Allopoda* sp. and *Oxycopis* sp.). Our specimens of *Asbolusmexicanusangularis* are the first reported from Maricopa County in Arizona and represent a roughly 50 mile (ca. 75 km) north-east range extension of that species. Many other species we report may represent additional notable range extensions, though the limited knowledge and digitised specimens from the region hinder more in-depth analyses.

**Figure 3.**
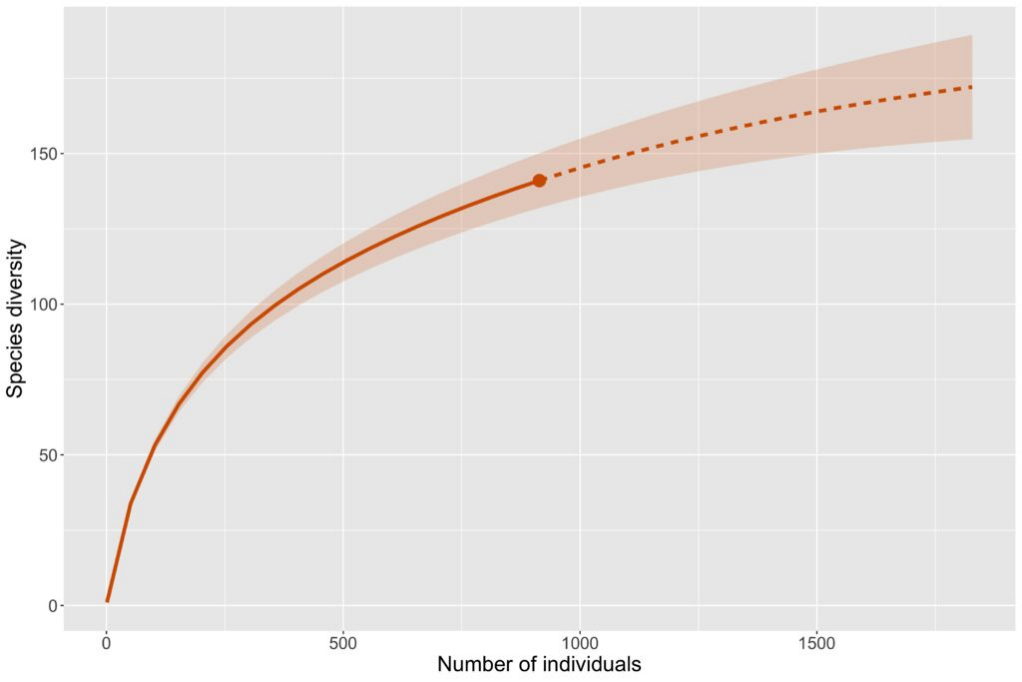
Species richness estimated by rarefaction and extrapolation for the Sand Tank Mountain Coleoptera fauna.

The actual number of Coleoptera species that inhabit the Sand Tank Mountains is surely much higher than what is recorded here. Based on our experience in the region, we presume this list is no more than 30% of the actual diversity and recommend future studies should focus on flower-feeding taxa and employ other trapping techniques, such as flight-intercept traps and Lindgren funnels. Estimating species richness using rarefaction and extrapolation (Fig. 3) estimates a total richness of 193 species which would mean we have sampled roughly 72% of the diversity so far. The lower estimate found in this analysis may be due to our employing similar collecting methods on all our trips. The estimate is perhaps a better reflection of the total number of species we could collect given the same techniques, while not accounting for taxa that diversifying techniques would add.

We would define the Coleoptera fauna of the Sand Tank Mountains as typical of the Lower Colorado River Basin of the Sonoran Desert. Many species we collected are typically found in arid low elevation regions of the State which is exemplified by the 30 species of Tenebrionidae collected which are highly diverse in such habitats. We postulate that the beetle fauna of the Sand Tank Mountains is likely similar to the fauna found throughout most of the low mountain ranges in the south-western portion of Arizona - but direct comparison is stifled by the lack of knowledge of those other mountain ranges.

### Collecting bias across ecoregions

Insect records and diversity for the ecoregions of Arizona are summarised in Fig. 4. The number of occurrence records are not very well correlated with the geographic area of the regions (Fig. 4a). When distinct collecting events are compared to geographic area (Fig. 4b), a trend of slightly more even distribution of sampling effort per area is observed. It seems clear that, relative to all the ecoregion in the State, the Madrean Archipelago (label 12.1.1 in figures) is disproportionately highly sampled, while the Arizona/New Mexico Plateau (label 10.1.7 in figures) is comparatively weakly sampled. This lack of correlation means that collecting efforts are not evenly distributed throughout space or between the different ecoregions.

### Collecting bias across mountain ranges

Insect records and collecting events for Arizona mountain ranges by geographic area are summarised in Fig. 5. In contrast to the data for ecoregions presented above, mountain ranges show a much less even distribution of collecting records. Both individual occurrence records by area (Fig. 5a) and collecting events by area (Fig. 5b) are highly skewed by a few very disproportionately well-collected mountain ranges and a large number of ranges with almost no sampling.

The most distant outlier by far is the Chiricahua Mountains (label 5 in Fig. 5) which are located in the extreme south-eastern corner of the State and represent 117,396 (40%) out of 296,421 total occurrence records which were mapped to all 81 mountain ranges examined here. This high sampling rate is, in large part, due to an active research station located within the range. The following four mountain ranges were also incredibly highly sampled, though nowhere near the sampling effort seen in the Chiricahuas. The Huachuca Mountains (label 6 in Fig. 5) are located along the Mexican border of Arizona and are home to many classical collecting localities and popular canyons, such as Ramsey Canyon, Miller Canyon and Carr Canyon. The Santa Rita Mountains (label 7 in Fig. 5) are just south of Tucson and have good access by roads, a university experimental station and are home to the very popular Madera Canyon. The Santa Catalina Mountains (label 9 in Fig. 5) are located just north of Tucson and are very easily accessed by paved roads from that city with classic collecting localities, such as Sabino Canyon and Mount Lemmon. The Atascosa/Pajarito Mountains (label 44 in Fig. 5) are also located along the middle of the Mexican border with Arizona and are home to the very well visited Pena Blanca Canyon and lake. Together, these five ranges represent 221,815 (75%) of the 296,421 total occurrence records from mountains reported here.

### Factors driving collecting bias

Analysis of the influence of proximity to roads, cities, airports and rivers is shown in Fig. 6. Proximity to roads was found to be the strongest factor of bias within our dataset, followed by proximity to cities and then airports. Proximity to rivers apparently has almost no effect on sampling bias. The underlying layer of roads was largely made up of paved, government-maintained roads and does not contain all smaller roadways that are often unpaved which provide access to most mountain ranges in Arizona. The analysis estimated both the weight of each biasing factor (Fig. 6a) and how that bias behaved by distance (Fig. 6b).

These biasing factors together generate a model of expected sampling frequency across Arizona. Fig. 7a shows this model rasterised across the State and Fig. 7b shows a heatmap of insect records overlaid on top. These visualisations demonstrate that, while proximity to population centres and roads are important, they clearly do not, alone, explain the distribution of insect collecting records across the State. In fact, the most heavily sampled Sky Islands in the south-western portion of the State are in areas of low expected collecting effort, while regions lying along major highways between population centres, such as the Interstate 10 corridor in central Arizona, are disproportionately less well collected.

### Species richness estimates

The disproportionate levels of data amongst mountain ranges discussed above demonstrate that it is too soon to accurately model insect diversity from occurrence records for most ranges. However, the Chiricahua Mountains are so disproportionately highly collected that they offer an important case study into what we can infer about insect diversity from occurrence records. Analysis of species richness for the Chiricahuas (Fig. 8) indicate that we are fairly far from reaching a plateau or accurate assessment of the actual taxonomic diversity. The preliminary estimate for all taxa (Fig. 8a) suggests 9,600 unique taxa are present, while more conservative estimate of species (Fig. 8b) suggests 6,500 species are present. Perhaps more important than the total numbers, both estimates suggest that current digitised data at best account for 70% of the actual diversity from those mountains.

Scaling up to ecoregions, species richness is similarly incompletely sampled by current collecting efforts (Fig. 9). The Madrean Archipelago, the proportionately highest sampled region by area (Fig. 4), boasts the largest recorded taxonomic diversity of the six ecoregions with just over 15,300 taxa which falls well short of a preliminary estimate of over 21,700. All ecoregions apparently require more than double the current sampling effort to begin to find a plateau and accurate species richness estimate.

Species richness estimates for the entire State of Arizona again fail to plateau with the available data (Fig. 10). Preliminary species richness estimates are much higher than those observed with all taxa (Fig. 10a) predicting roughly 36,000 total taxa and species-only data (Fig. 10b) predicting just over 21,600 species. As with the Chiricahua Mountains and ecoregions discussed above, both Arizona richness estimates imply that the current data only represent around 70% of predicted diversity and similarly demonstrate that online data will need to be greatly expanded before accurate estimates can be made.

## Discussion

### Species richness estimates

All rarefaction and extrapolation estimates of species (or taxonomic) richness failed to plateau and provided very similar results that only 70% of the full estimated species richness were observed. As all the analyses across the three scales explored here gave these similar proportional results, it seems clear that the estimates are strongly affected by incomplete sampling. We again urge readers to be cautious with the absolute numbers presented here. However, given our knowledge of the Sand Tank Mountains Coleoptera study and its limitations along with the slopes of all rarefaction curves, the species richness estimates presented here seem to be extremely conservative counts and might be useful as a lowest-end predictor of what the true diversity is. We did not assess the diversity of collecting techniques represented in our dataset. This may mean that we are underestimating total insect taxa, even at larger scales, due to inadequate sampling techniques.

### Additional factors driving collecting bias

The analyses presented here clearly demonstrate that, according to available data, insect collecting has not been done evenly throughout the State. The underlying factors that drive the biases seen in the data are likely numerous and difficult to fully ascertain. We hypothesise that two of the primary drivers are habitat accessibility and social interactions.

Habitat access for insect collectors is very important and has many facets. Proximity to roads and populations centres is clearly important, but not the only limiting factor and not all cities and roads are the same. For example, the Chiricahua Mountains have roads accessible to passenger cars that go to the highest elevations. The Mountains are almost entirely on public lands and there are nearby towns with accommodation and stores, as well as a popular research station. In contrast, all sites visited in the Sand Tank Mountains involved rugged back-country roads requiring high clearance and four-wheel drive vehicles in areas where it is unlikely to encounter other people in the event of an emergency. Habitat access is not equal at the large scale of ecoregions either. The majority of the Madrean Archipelago is covered by public lands (U.S. Forest Service and Bureau of Land Management) while large swathes of the Sonoran Desert and especially the Arizona/New Mexico Plateaus regions are Native American Reservations. Different sovereign tribal lands, private property and various public land management agencies all have unique regulations and permitting processes which affect collecting insects. The Mohave Basin and Range ecoregion is an interesting example of how these factors interact where most of the lands are public, but the terrain is very rugged and roads and population centres are limited which is likely why there are so few insect records from the region even though there are no major permitting restrictions.

The Patagonia Picnic Table Effect (Laney et al. 2021), named after the town of Patagonia, Arizona, is a term from the birdwatching community to describe how one sighting of a rare species leads to increased birdwatching effort in the immediate region. The equivalent in the insect world would be one collector finding a very rare or charismatic species which prompts future collectors to either go to the same locality to collect either that same species for themselves or in hopes that it might also produce a rare species of their own group of interest. Laney et al. (2021) analysed 10 years of birdwatching data and demonstrated that there is an increase of activity following the initial discovery, but there was no increased likelihood to find additional rare species in that area compared to any other. It seems clear that, despite its potential lack of utility in rare species documentation, it is a social phenomenon in naturalist communities which may also contribute to uneven insect collecting thoughout the state.

### Recommendations for future collecting

The full scale of insect diversity has been under-documented for the State of Arizona, its constituent ecoregions or even its most popular mountain ranges, at least in available online data. It is important that the entomological community continues to survey for and collect insects everywhere in the State. Continuing and increasing efforts to mobilise specimen data from natural history collections also remains a high priority and will likely help to account for many species which are not currently represented in online data. It is estimated that not more than 5% of specimens in insect collections of the United States have been fully digitised (Cobb et al. 2019). It is possible that some of the biases found in our dataset will be corrected as this proportion increases and it will be very interesting to see what will happen to species accumulation curves as the data increase. We would recommend that gap analyses be done on digitised insect data when the number of records approximately doubles from its current state since none of the species richness estimation curves reported here approached an asymptote.

We urge collectors to make a concerted effort to go to new places and consider targeting specifically undercollected regions and mountain ranges. Small and targeted studies can exponentially improve our understanding of Arizona insect fauna and are likely the best way to increase knowledge of species distributions and may be crucial to understanding the entire State fauna. Our example of the Sand Tank Mountains beetles highlights how a modest collecting effort can still provide new occurrence records for species from the State and report on new localities for taxa that are otherwise considered rare in collections. We do not recommend that collectors avoid the classic and popular sites; indeed, we still need to sample those, but we would advocate that entomologists consider dividing their time in the field and only spend part of their efforts in the well-known habitats and spend the next day somewhere new.

The paucity of insect data from so many mountain ranges in the State strongly limit our ability to adequately protect and conserve insect biodiversity. Entomologists and insect collections should partner with local, State and federal land management agencies to increase insect sampling throughout the State. Increasing partnerships and professional connections with tribal nations within the State are also strongly recommended. Opportunites for occurrence-data driven estimates for species diversity are in their infancy, even for a biodiversity hotspot that is accessible and popular. Nevertheless, the growing availability of occurrence data is an important resource to continue to develop to understand the diversity and distributions of insects.

## Supplementary Material

0A29E9FB-A9B6-550B-8E0F-58C1F5748A76 10.3897/BDJ.11.e101960.suppl1 Supplementary material 1 Arizona insect occurrence data annotated by which ecoregion or mountain range they occur within Data type occurrences Brief description Zip archive of two occurrence recordsets for Arizona insects. The first file contains occurrence records annotated by which ecoregion they fall within and the second file contains annotated records by which mountain range they fall within. File: oo_777372.zip https://binary.pensoft.net/file/777372 M.A. Johnston

67767A50-A46D-5AD6-8037-12364431B6E7 10.3897/BDJ.11.e101960.suppl2 Supplementary material 2 Arizona ecoregion data Data type Geographical and biodiversity metrics of ecoregions Brief description This table includes data for all Arizona ecoregions including their geographic size and the number of insect records, collecting events and taxa found within them. File: oo_809256.csv https://binary.pensoft.net/file/809256 M.A. Johnston

C849E347-8C8F-5F75-B79E-C297E73DBD1D 10.3897/BDJ.11.e101960.suppl3 Supplementary material 3 Arizona mountain range data Data type Geographic and biodiversity metrics for mountain ranges Brief description This table includes information about the tallest mountain ranges in Arizona. A polygon in WKT format, the geographic area, prominence and height of each mountain range is given. Totals for insect occurrence records, collection events and taxa are also given. File: oo_777374.csv https://binary.pensoft.net/file/777374 M.A. Johnston

B1AD592F-633E-52FC-BC3E-65E235D27A66 10.3897/BDJ.11.e101960.suppl4 Supplementary material 4 Sand Tank Coleoptera specimen records Data type occurrences Brief description Darwin Core Archive of new records File: oo_711180.zip https://binary.pensoft.net/file/711180 M.A. Johnston

20F9779E-8E23-5849-ACA1-2935A775DFCA 10.3897/BDJ.11.e101960.suppl5 Supplementary material 5 Data analysis and figure generation script Data type R script used for data analysis and for generating all the statistical figures used in this paper. File: oo_806970.R https://binary.pensoft.net/file/806970 M. Andrew Johnston

DDC6B965-787C-58CB-987F-A1365D78AF94 10.3897/BDJ.11.e101960.suppl6 8098911 Supplementary material 6 Additional analyses for normality and log-transformed data Data type statistical graphs Brief description This file contains graphical analyses to test the Arizona insect occurrence dataset for normality across mountain ranges and Ecoregions. Additional graphs of log-transformed data are included for untransformed analyses presented in the main text. File: oo_846719.pdf https://binary.pensoft.net/file/846719 M. Andrew Johnston

## Figures and Tables

**Figure 1a.**
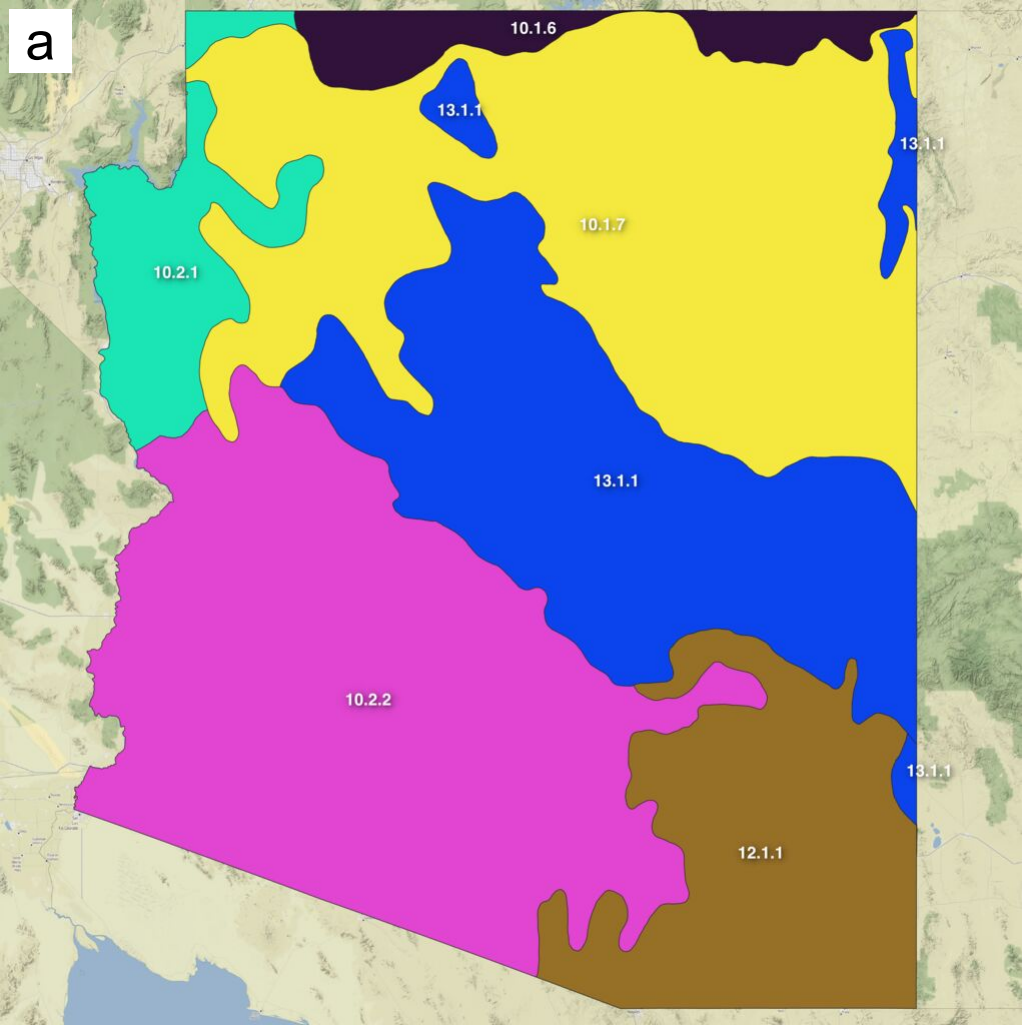
Ecoregions of Arizona. Labels correspond to ecoregion codes with matching names as follows: 10.1.6 = Colorado Plateaus; 10.1.7 Arizona/New Mexico Plateau; 10.2.1 = Mojave Basin and Range; 10.2.2 = Sonoran Desert; 12.1.1 = Madrean Archipelago; 13.1.1 = Arizona/New Mexico Mountains.

**Figure 1b.**
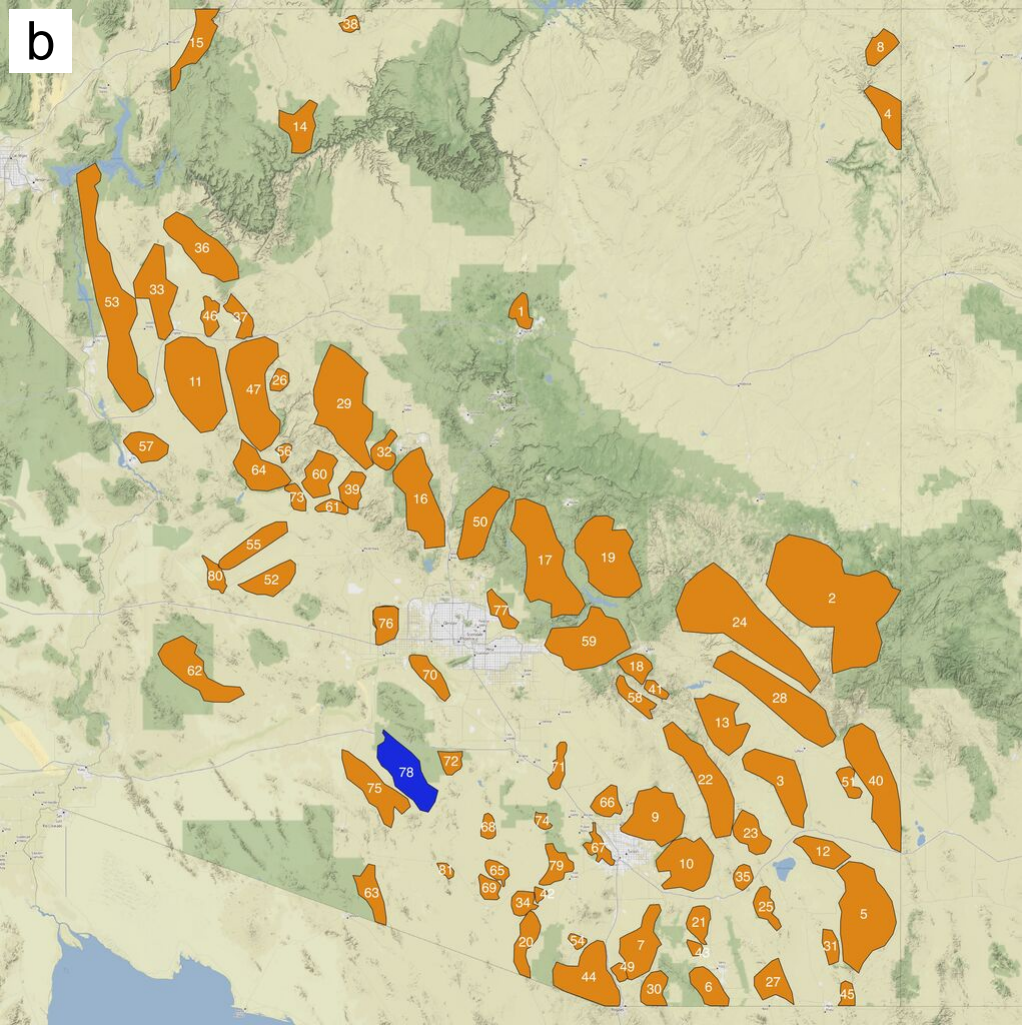
Arizona mountain ranges. Labels refer to mountain range details in Suppl. material 3. The Sand Tank Mountains, number 78, are shaded in blue.

**Figure 1c.**
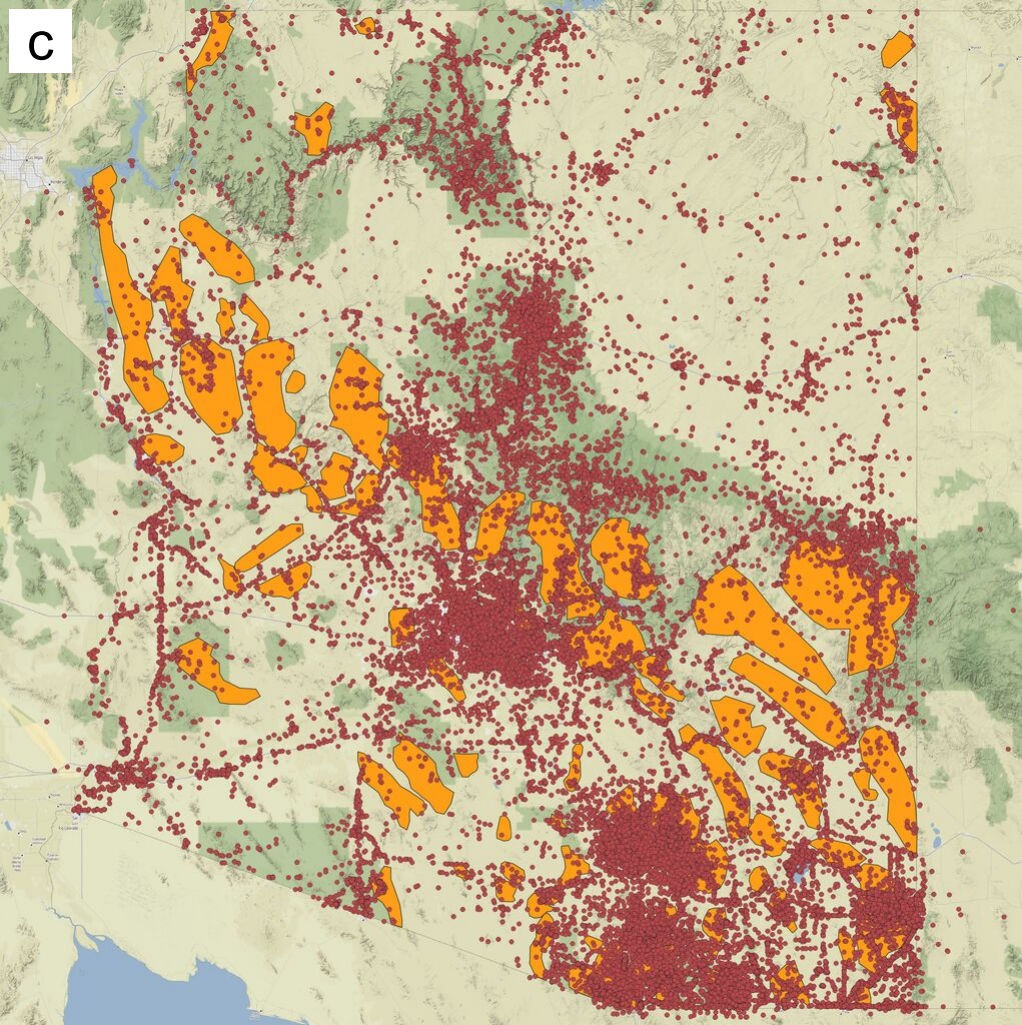
Georeferenced insect records from Arizona. Shapes underneath are outlines of mountain ranges shown in B.

**Figure 1d.**
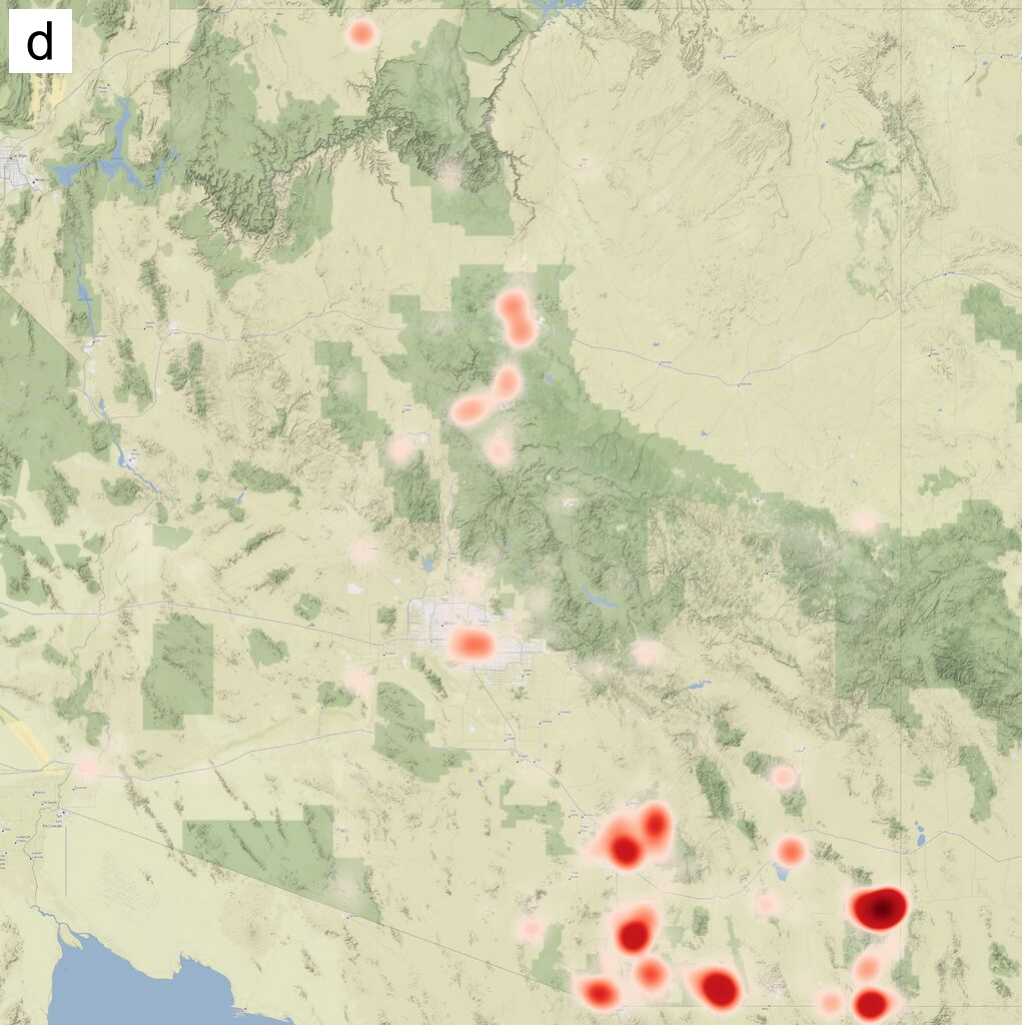
Heatmap of georeferenced insect records from Arizona.

**Figure 2a.**
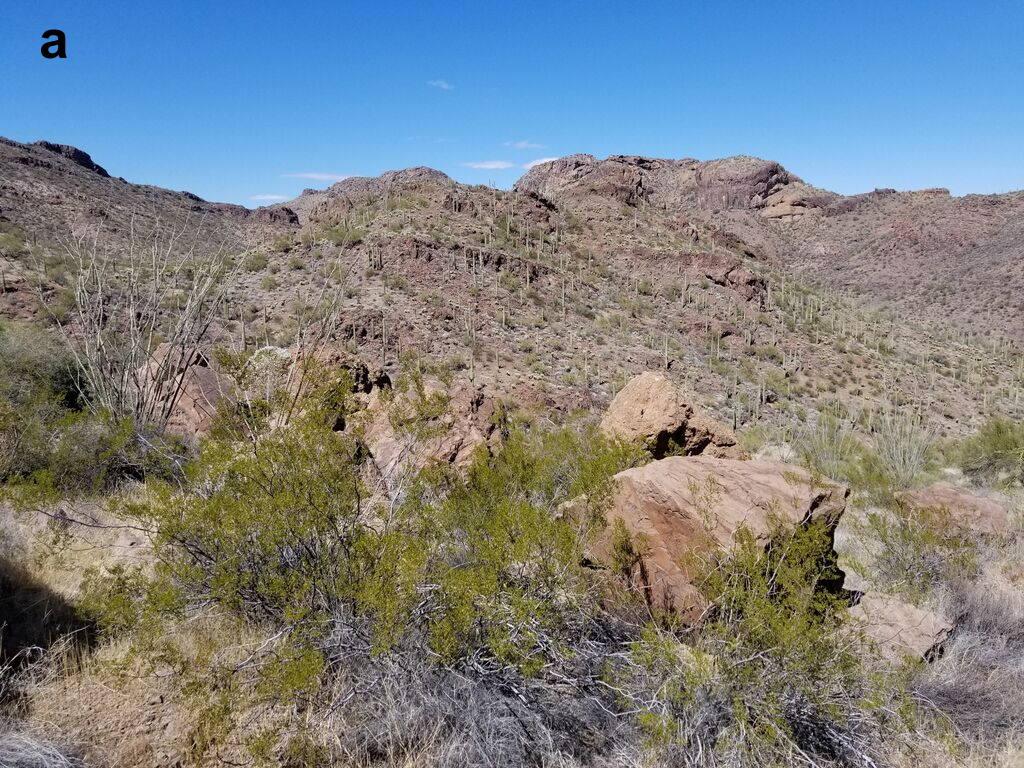
Typical habitat and vegetation of north-facing slopes in the Sand Tank Mountains.

**Figure 2b.**
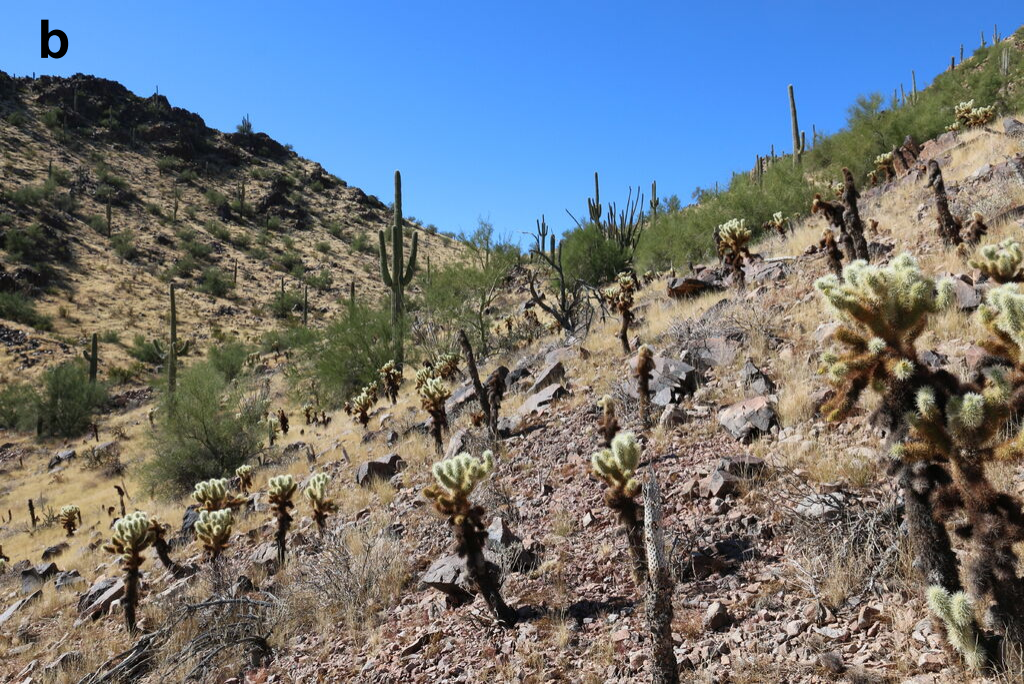
Typical habitat and vegetation of south-facing slopes in the Sand Tank Mountains.

**Figure 2c.**
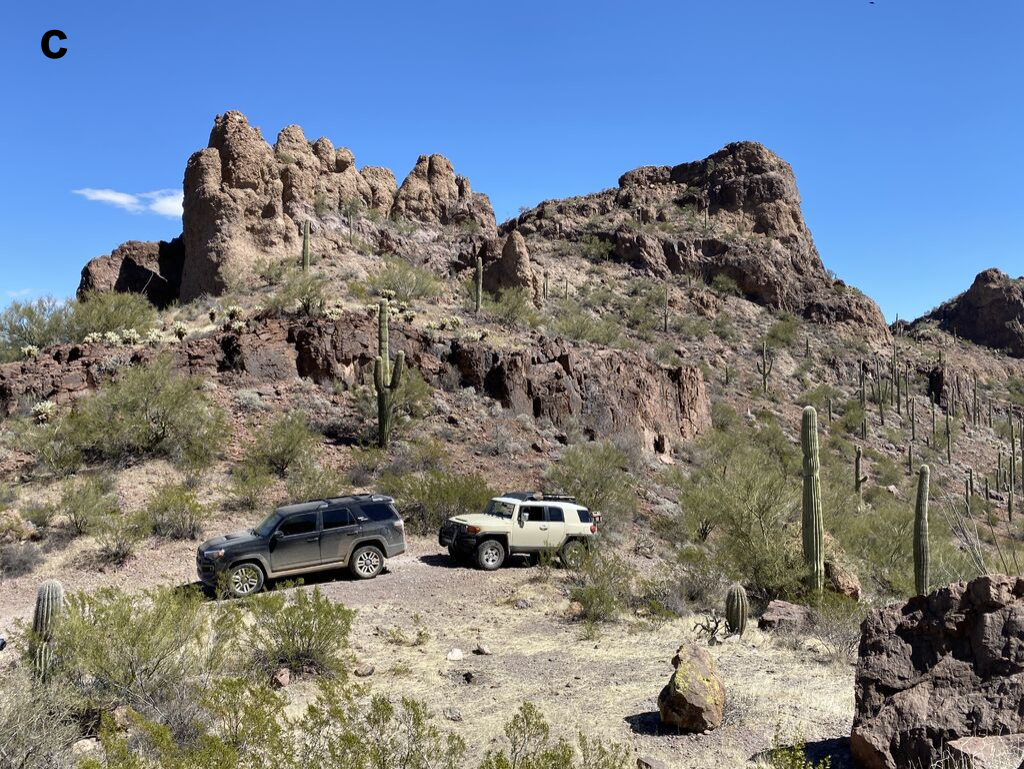
Mountain pass with 4-wheel-drive trail leading into the valley containing Bender Spring, one of the main sampling sites we visited in the Sand Tank Mountains.

**Figure 2d.**
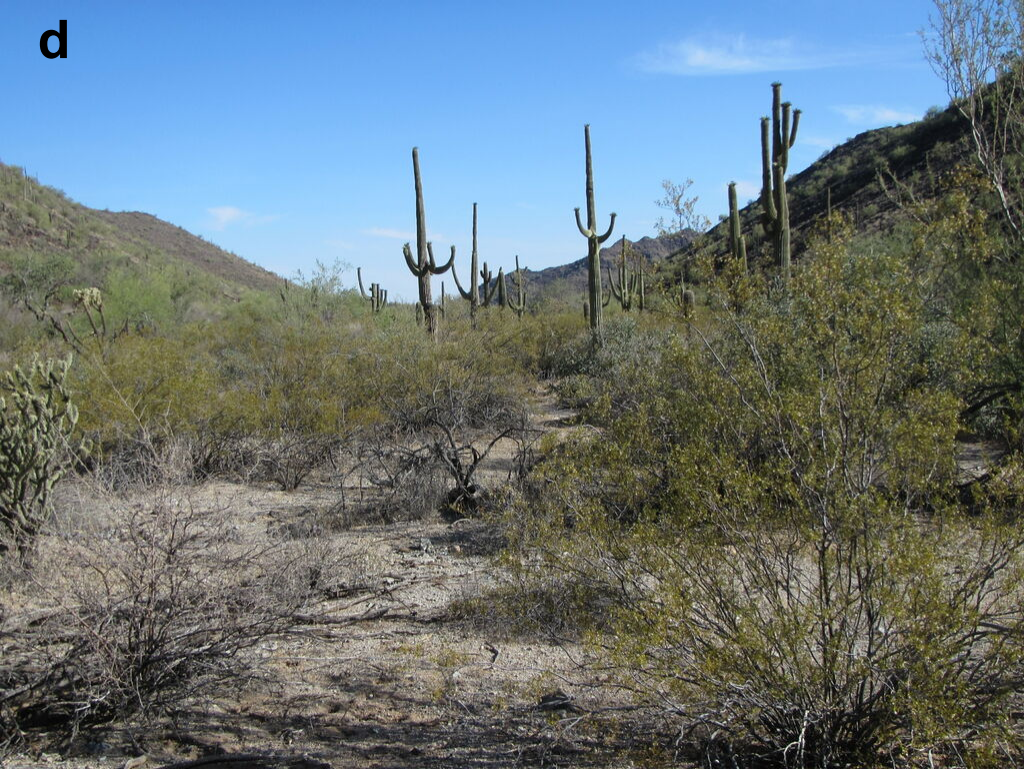
Sandy valley habitat which was one of the sampling sites we visited in the Sand Tank Mountains.

**Figure 4a.**
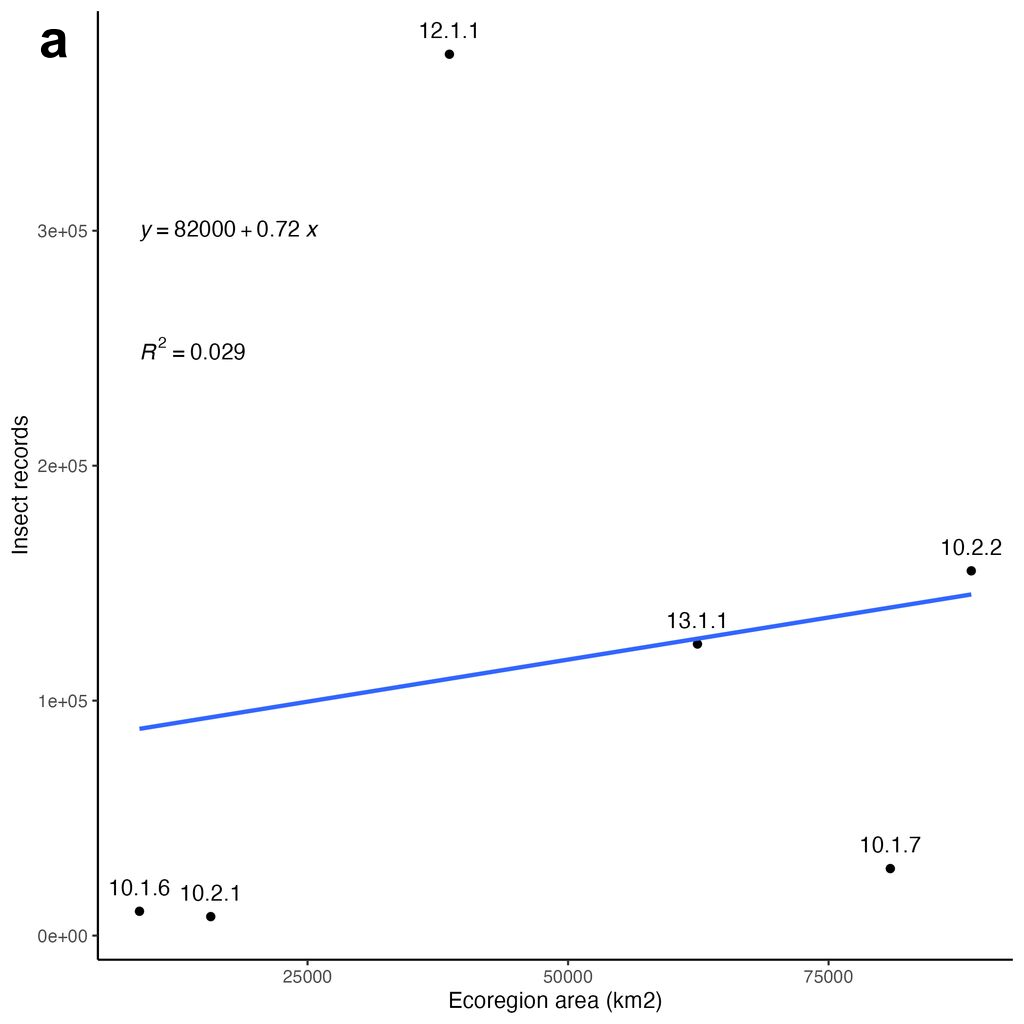
Digitised insect records by geographic area (km^2^) of ecoregions in Arizona.

**Figure 4b.**
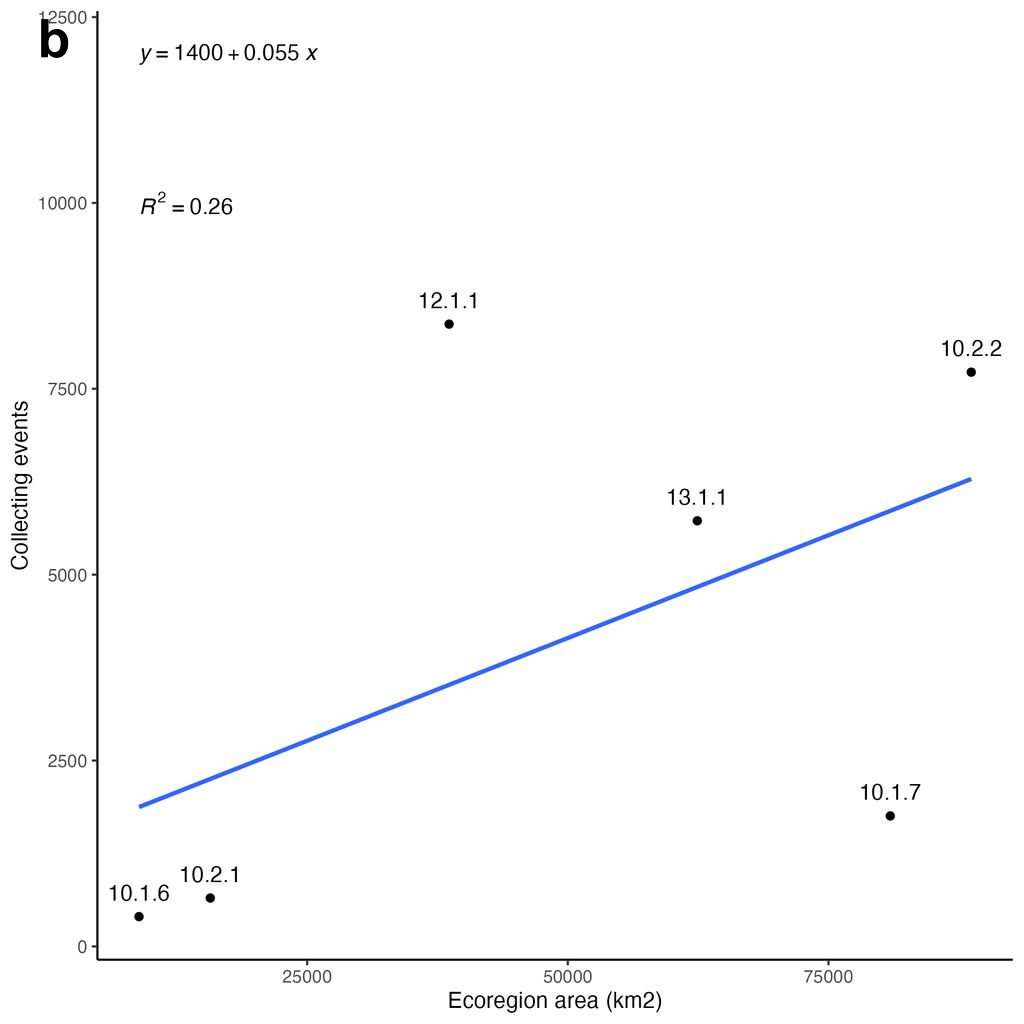
Collecting events by geographic area (km^2^) of ecoregions in Arizona. A single collecting event corresponds to all specimens with the same collector (DWC:recordedBy) and date (DWC:day, month, year).

**Figure 5a.**
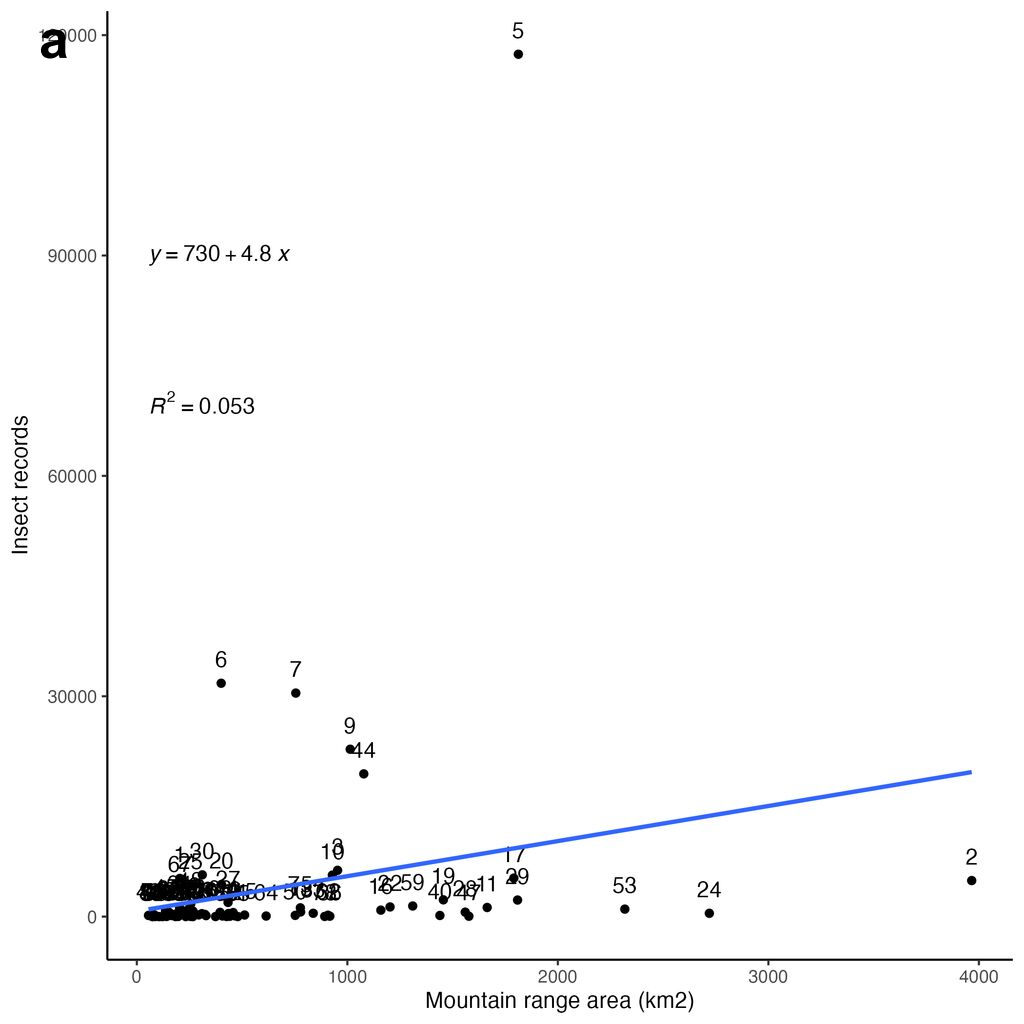
Digitised insect records by geographic area (km^2^) of mountain ranges in Arizona.

**Figure 5b.**
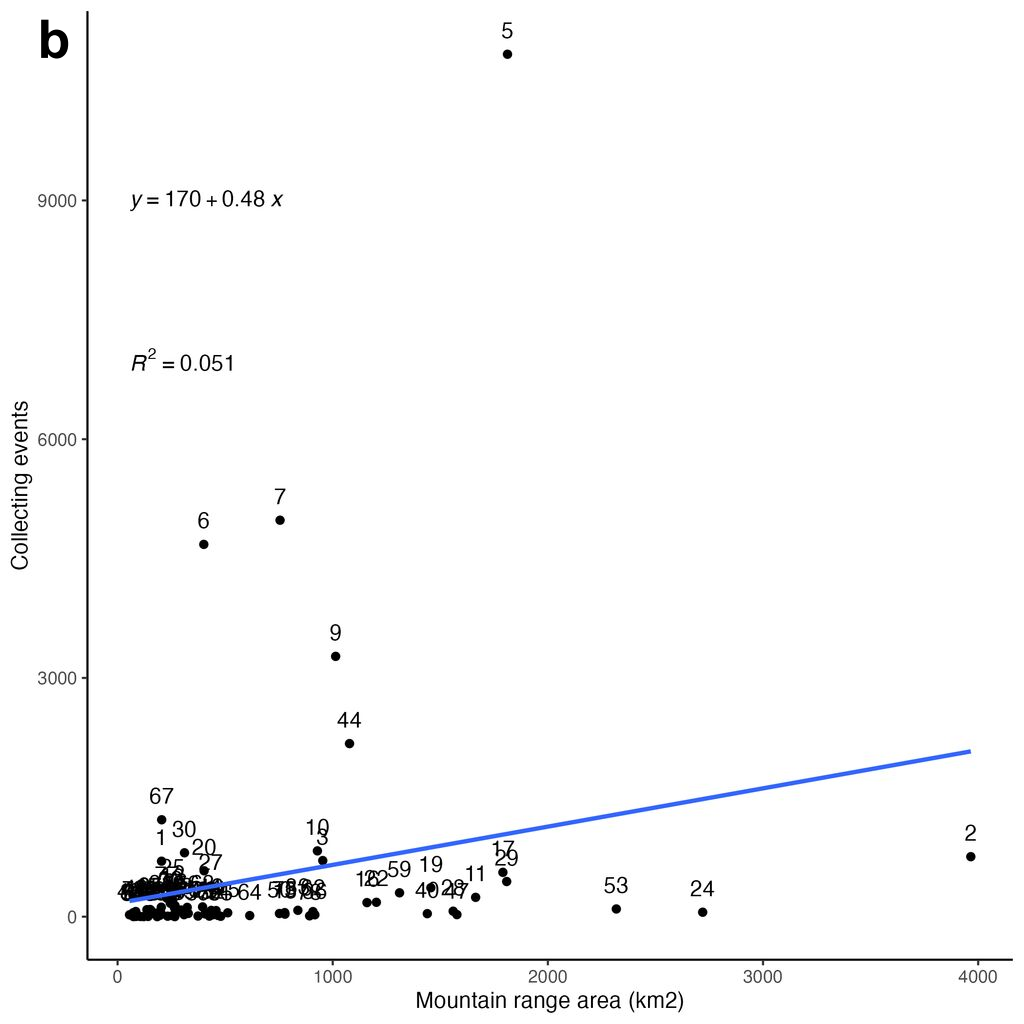
Collecting events by geographic area (km^2^) of mountain ranges in Arizona. A single collecting event corresponds to all specimens with the same collector (DWC:recordedBy) and date (DWC:day, month, year).

**Figure 6a.**
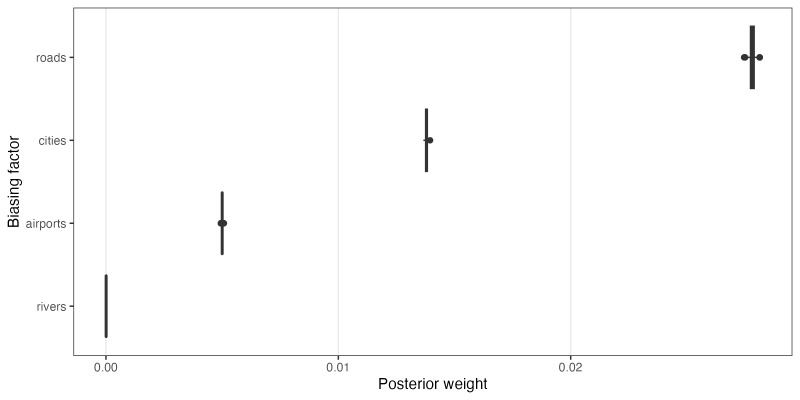
Inferred posterior weight of each biasing factor. Each is represented by a narrow range, likely due to the size of our occurrence dataset.

**Figure 6b.**
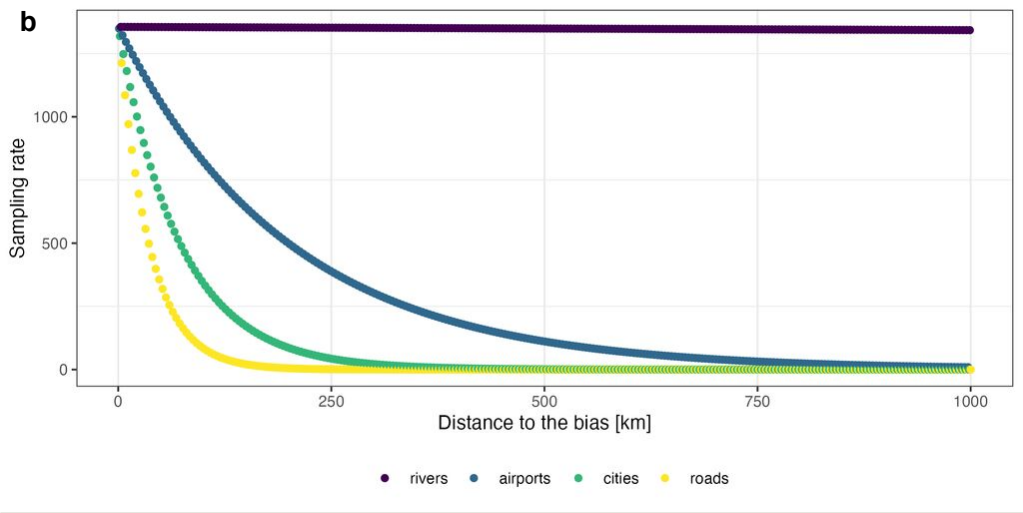
Estimated sampling rate as a function of distance in kilometres from the biasing factor.

**Figure 7a.**
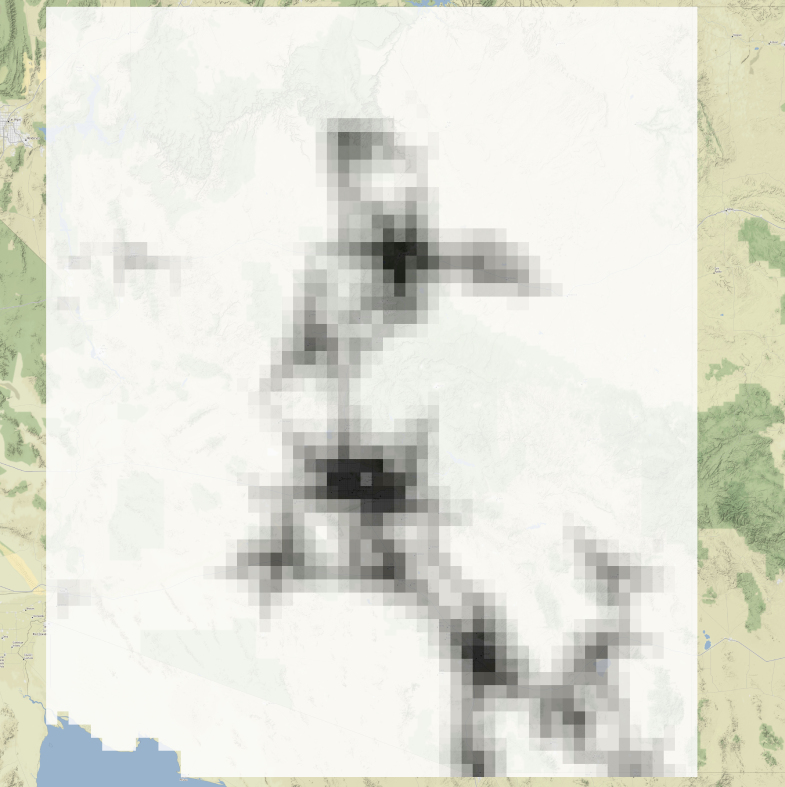
Expected sampling effort given model of collecting bias. Darker hues represent higher expected sampling, while lighter hues represent lower expected sampling.

**Figure 7b.**
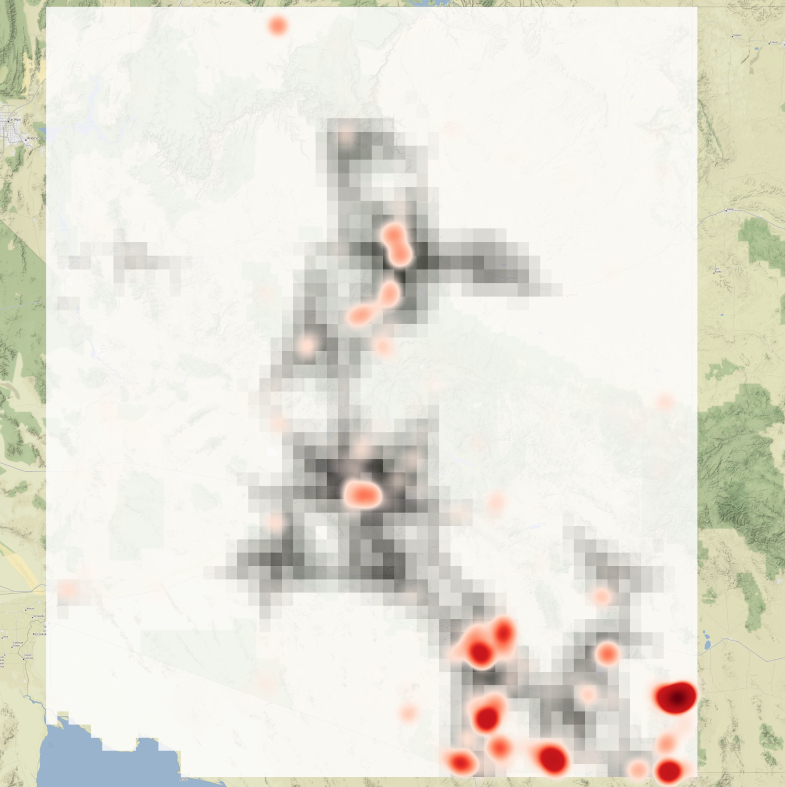
Expected sampling effort overlaid with heatmap of insect occurrence records showing actual sampling effort compared to the model.

**Figure 8a.**
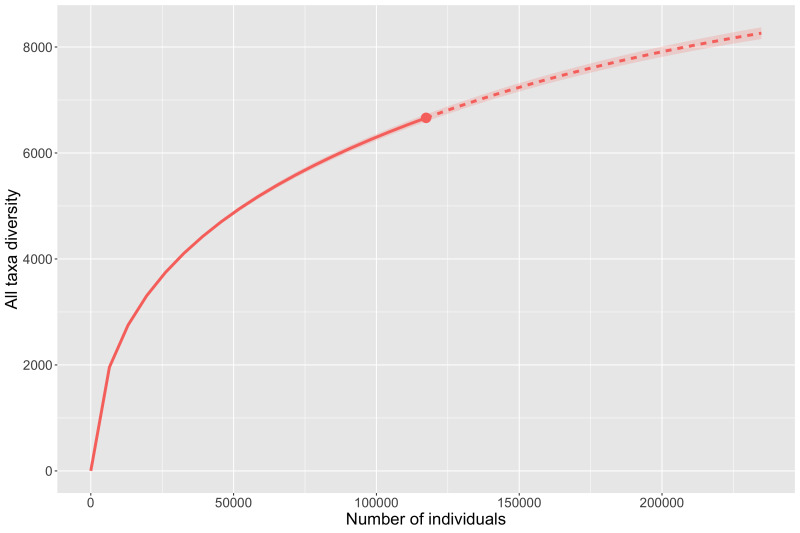
Rarefaction and estimation curve for all taxa from all ranks for the Chiricahua Mountains (6,663 distinct taxon names observed).

**Figure 8b.**
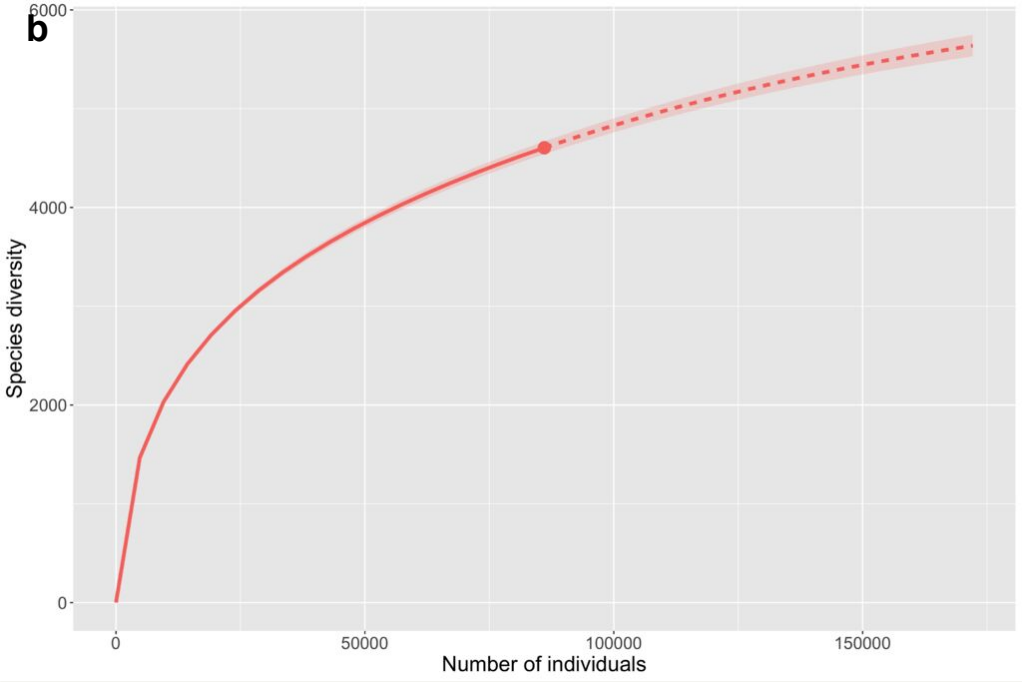
Rarefaction and estimation curve for species level taxa for the Chiricahua Mountains (4,604 distinct taxon names observed).

**Figure 9a.**
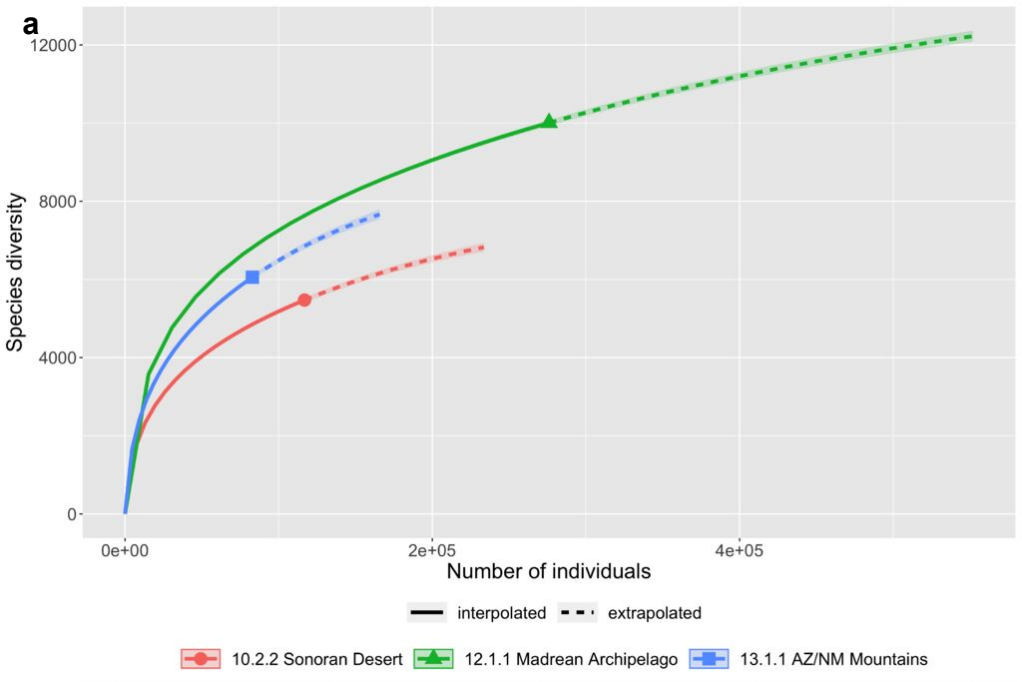
Sonoran Desert, Madrean Archipelago and Arizona/New Mexico Mountains ecoregions.

**Figure 9b.**
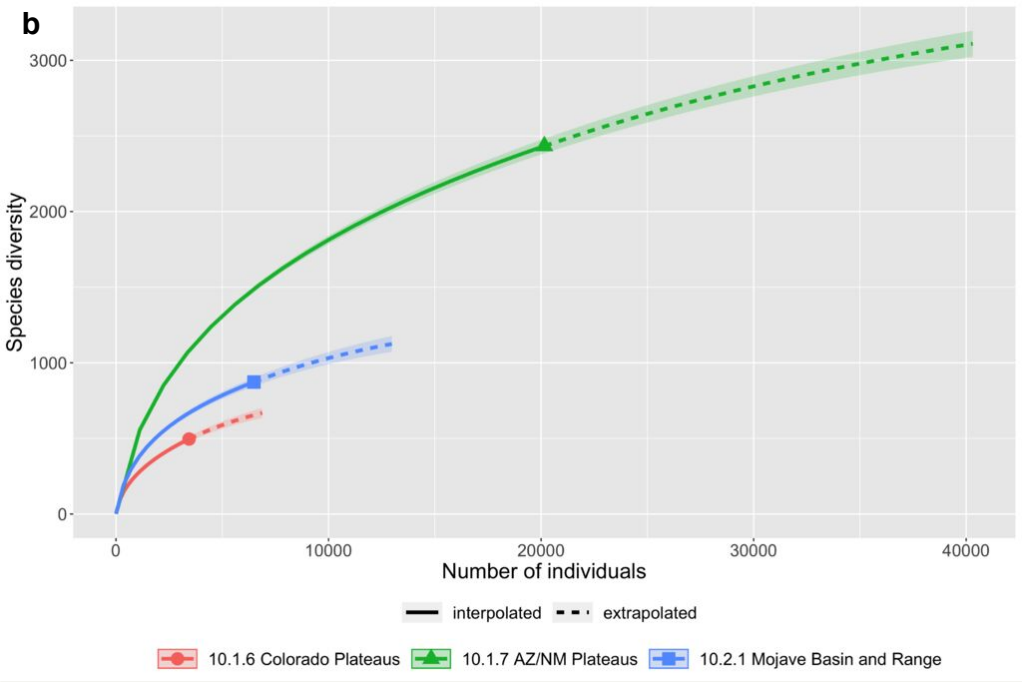
Colorado Plateaus, Arizona/New Mexico Plateaus and Mojave Basin and Range ecoregions.

**Figure 10a.**
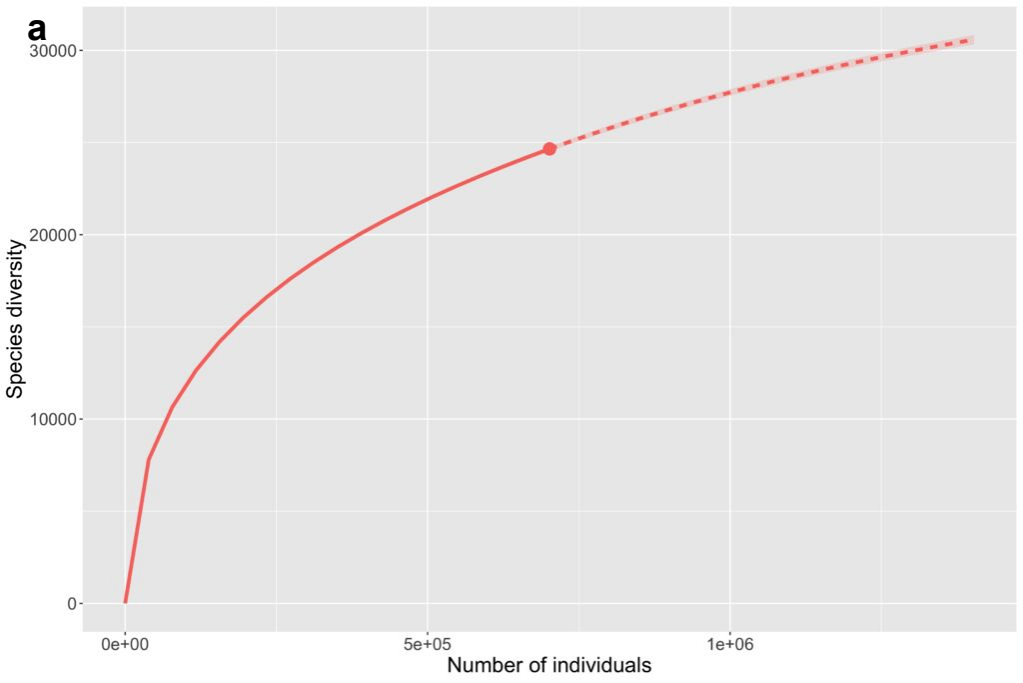
Rarefaction and estimation curve for all taxa from all ranks for Arizona (24,651 distinct taxon names observed).

**Figure 10b.**
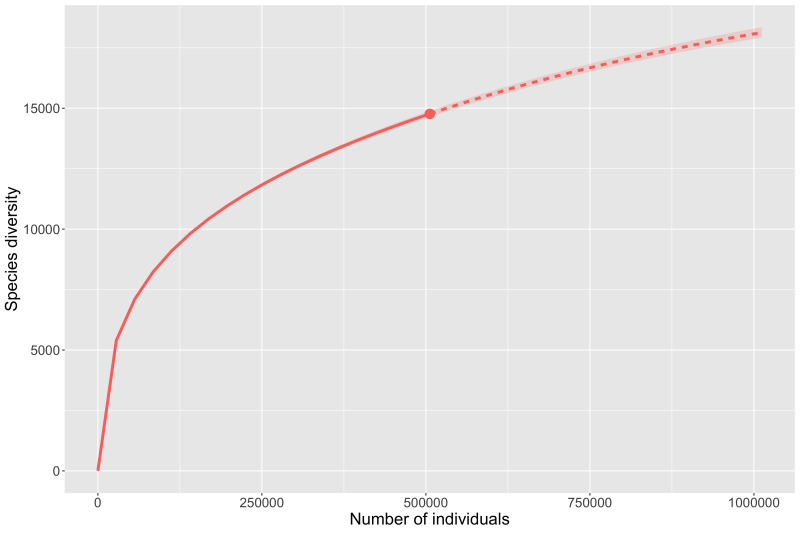
Rarefaction and estimation curve for all species level taxa for Arizona (14,769 distinct species names observed).
