# Supplementary material for: Insect collecting bias in Arizona with a preliminary checklist of the beetles from the Sand Tank Mountains
Source: Biodivers Data J. 2023 Jun 28;11:e101960. doi: 10.3897/BDJ.11.e101960 (PMC10323768; doi:10.3897/BDJ.11.e101960)
Supplement: Supplementary material 6 — Additional analyses for normality and log-transformed data [file bdj-11-e101960-s006.pdf]

Additional analyses for normality and log-transformed data

Normality was assessed for each dataset for mountain ranges and ecoregions. A Shapiro-Wilk test was performed (significant p-value meaning the dataset differs from a normal distribution) along with a box plot, histogram, and plotting of residuals. These are represented in the graphs below:

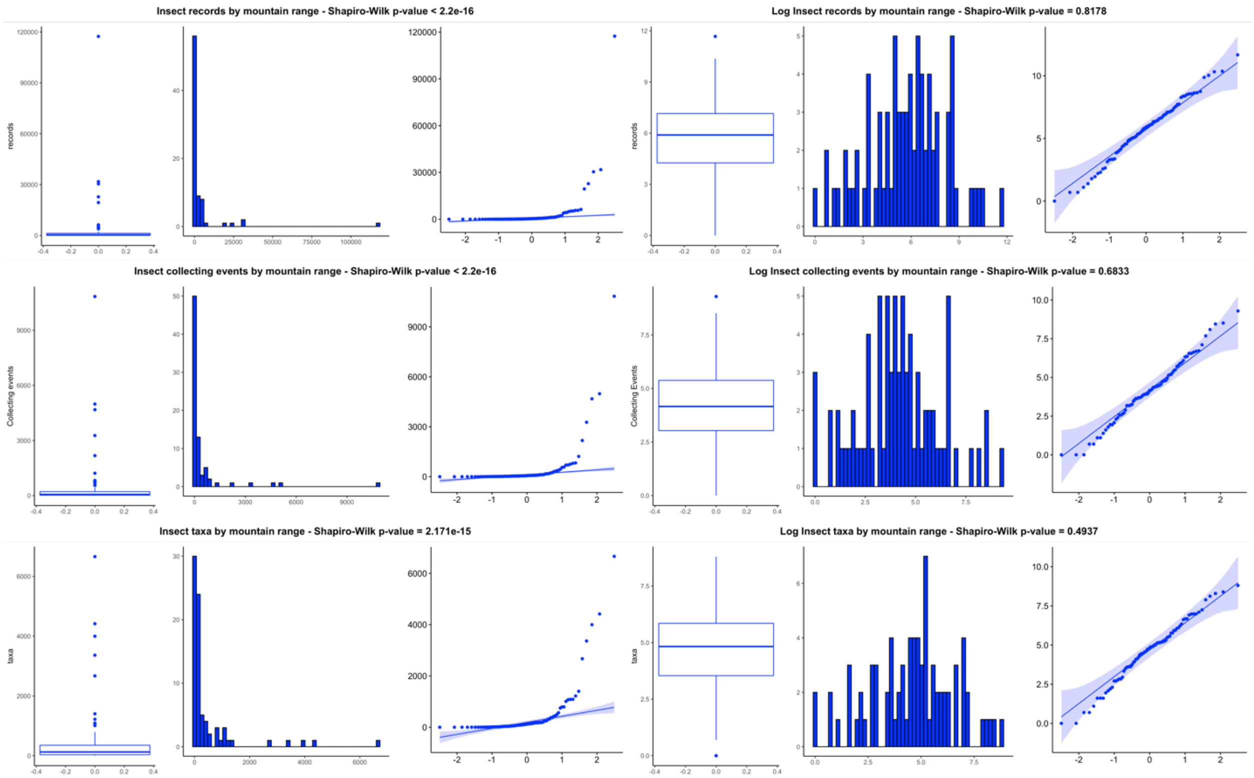

Additional plots of log transformed data by ecoregion and mountain range area:

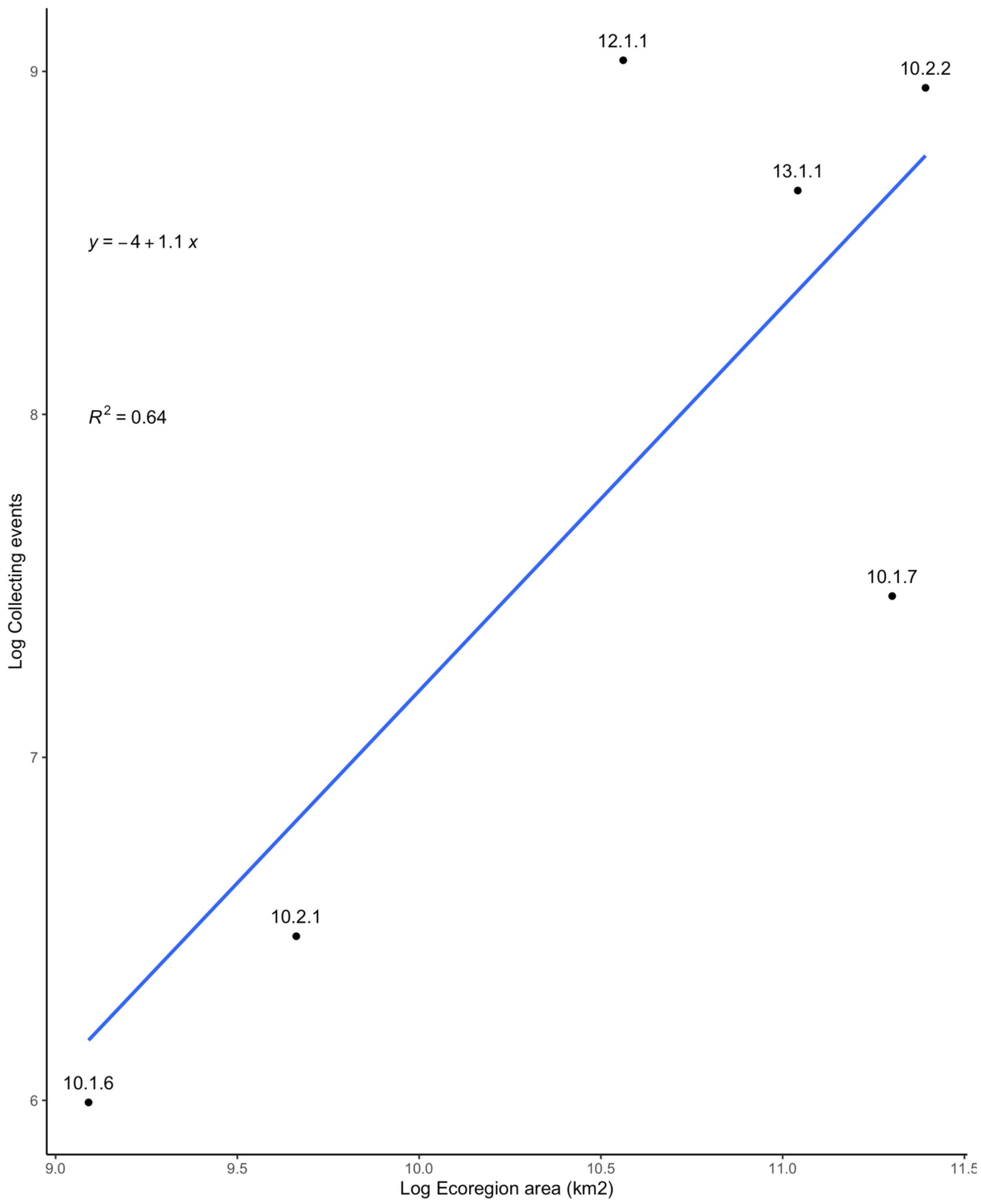

12.1.1
10.2.2
13.1.1
y = −4 + 1.1 x
R2 = 0.64
10.1.7
10.2.1
10.1.6
9
8
7
6
Log Collecting events
9.0
9.5
10.0
10.5
11.0
11.5
Log Ecoregion area (km2)

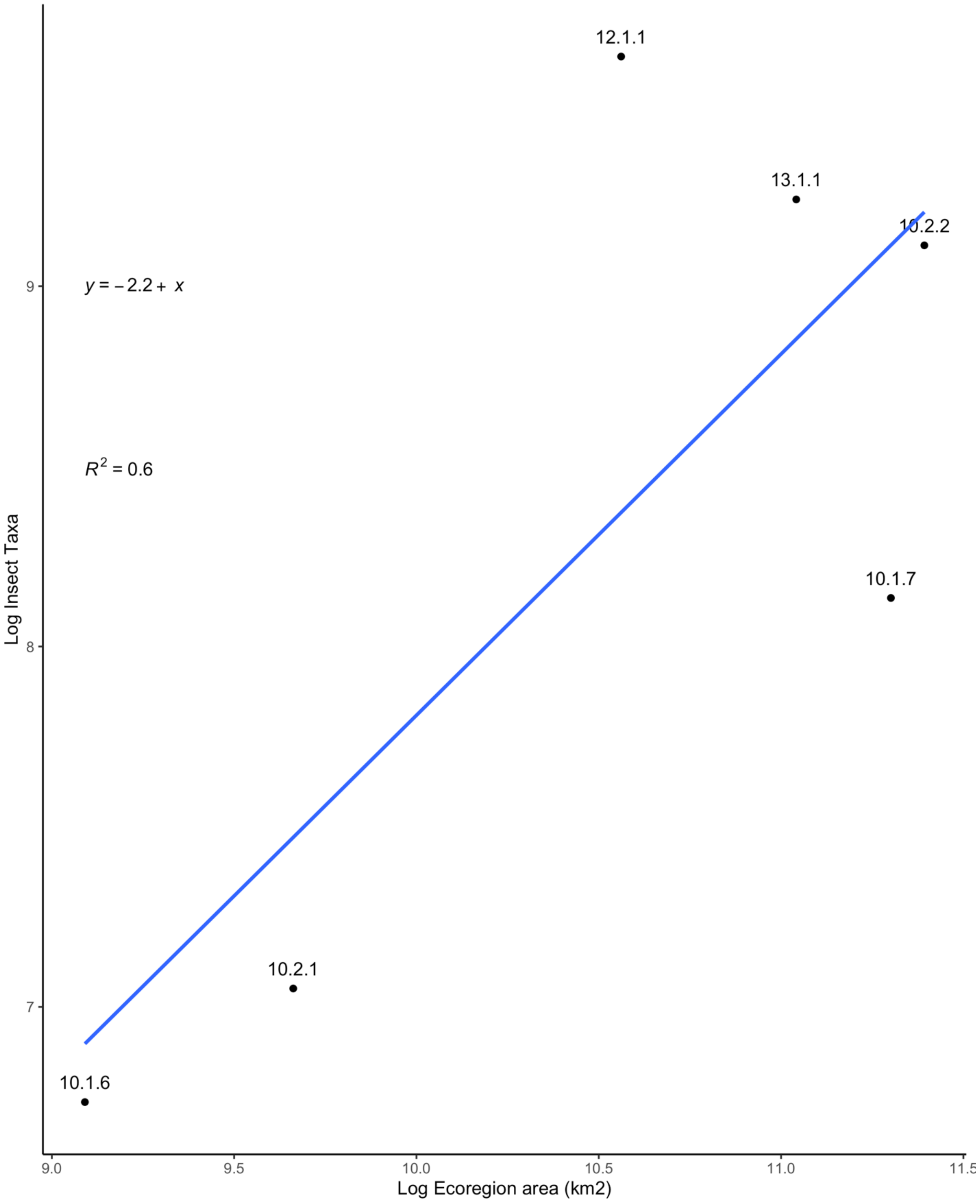

12.1.1
13.1.1
10.2.2
$y = -2.2 + x$
$R^2 = 0.6$
10.1.7
10.2.1
10.1.6
9
8
7
Log Insect Taxa
9.0
9.5
10.0
10.5
11.0
11.5
Log Ecoregion area (km2)

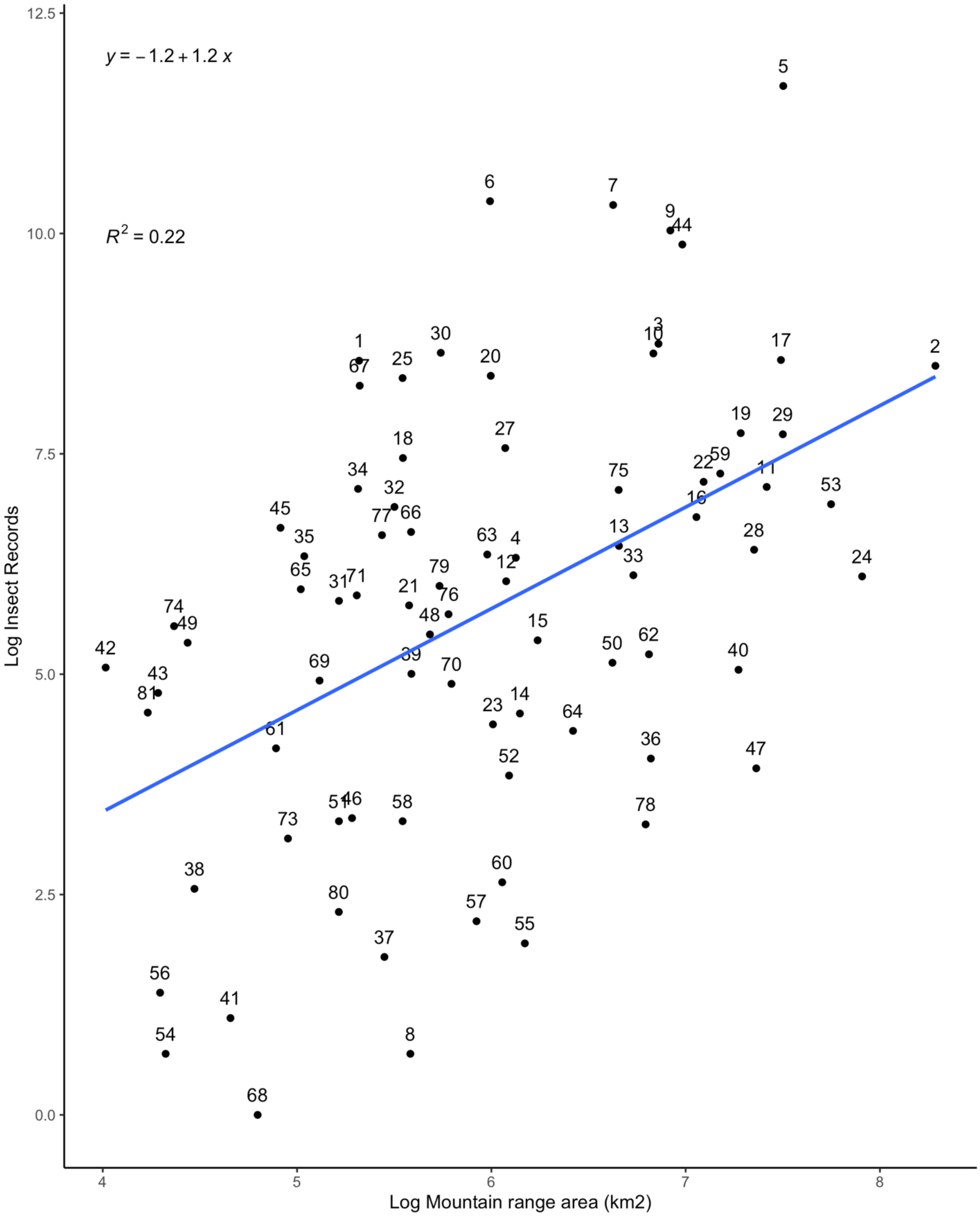

$y = -1.2 + 1.2\,x$
$R^2 = 0.22$
Log Insect Records
Log Mountain range area (km2)
0.0
2.5
5.0
7.5
10.0
12.5
4
5
6
7
8

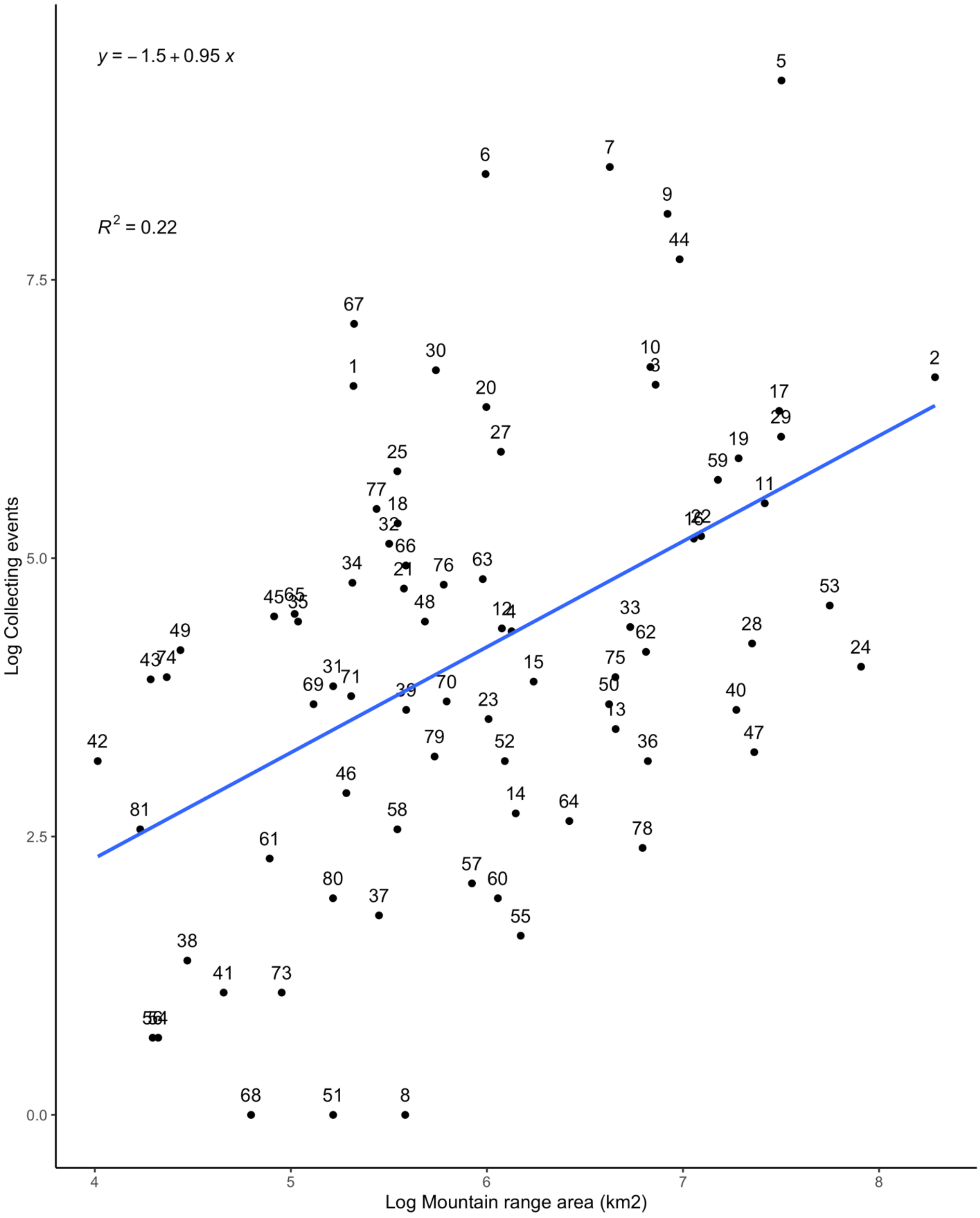

$y = -1.5 + 0.95\,x$
$R^2 = 0.22$
Log Collecting events
Log Mountain range area (km2)
0.0
2.5
5.0
7.5
4
5
6
7
8

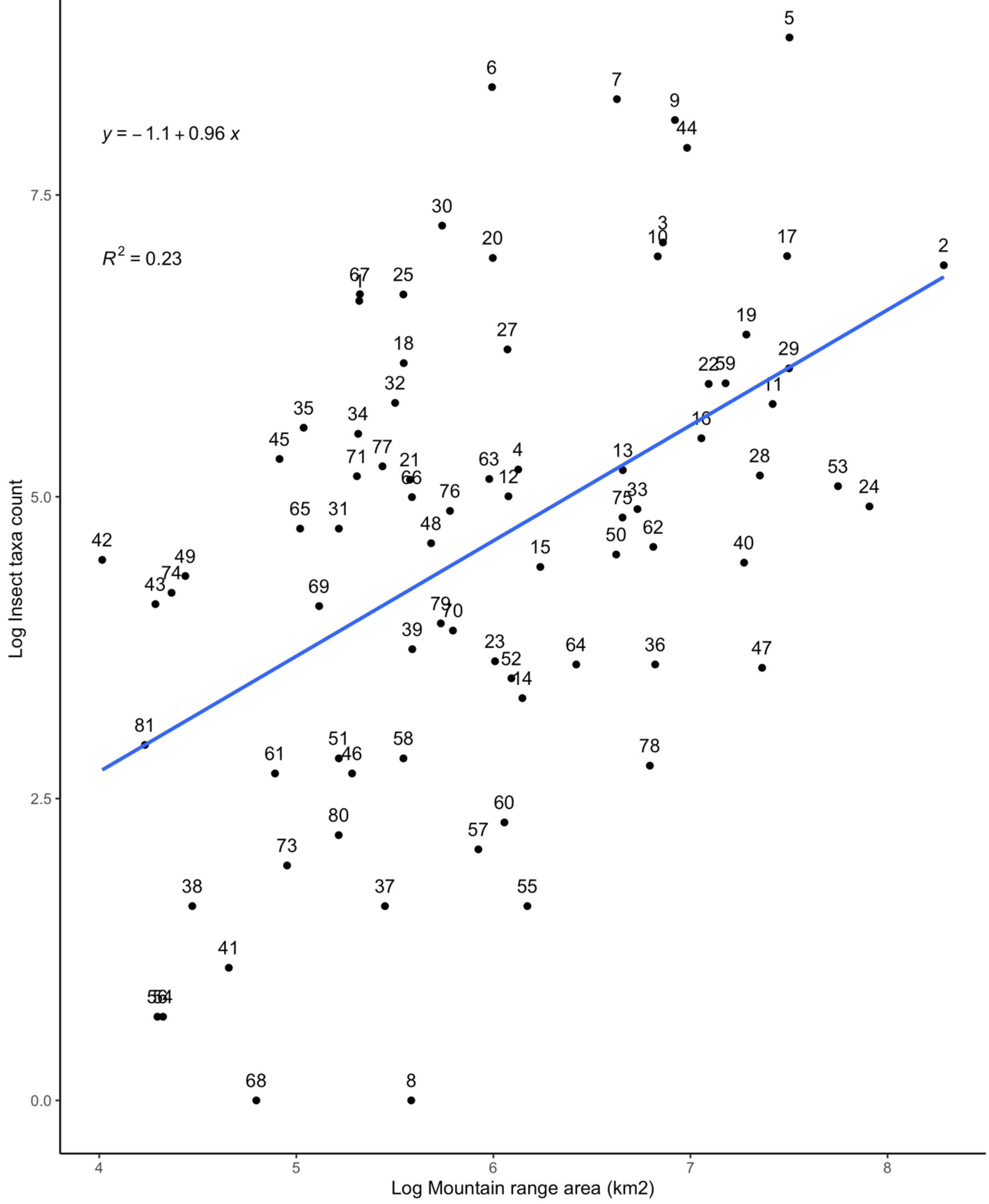

$y = -1.1 + 0.96\,x$
$R^2 = 0.23$
Log Insect taxa count
Log Mountain range area (km2)
0.0
2.5
5.0
7.5
4
5
6
7
8

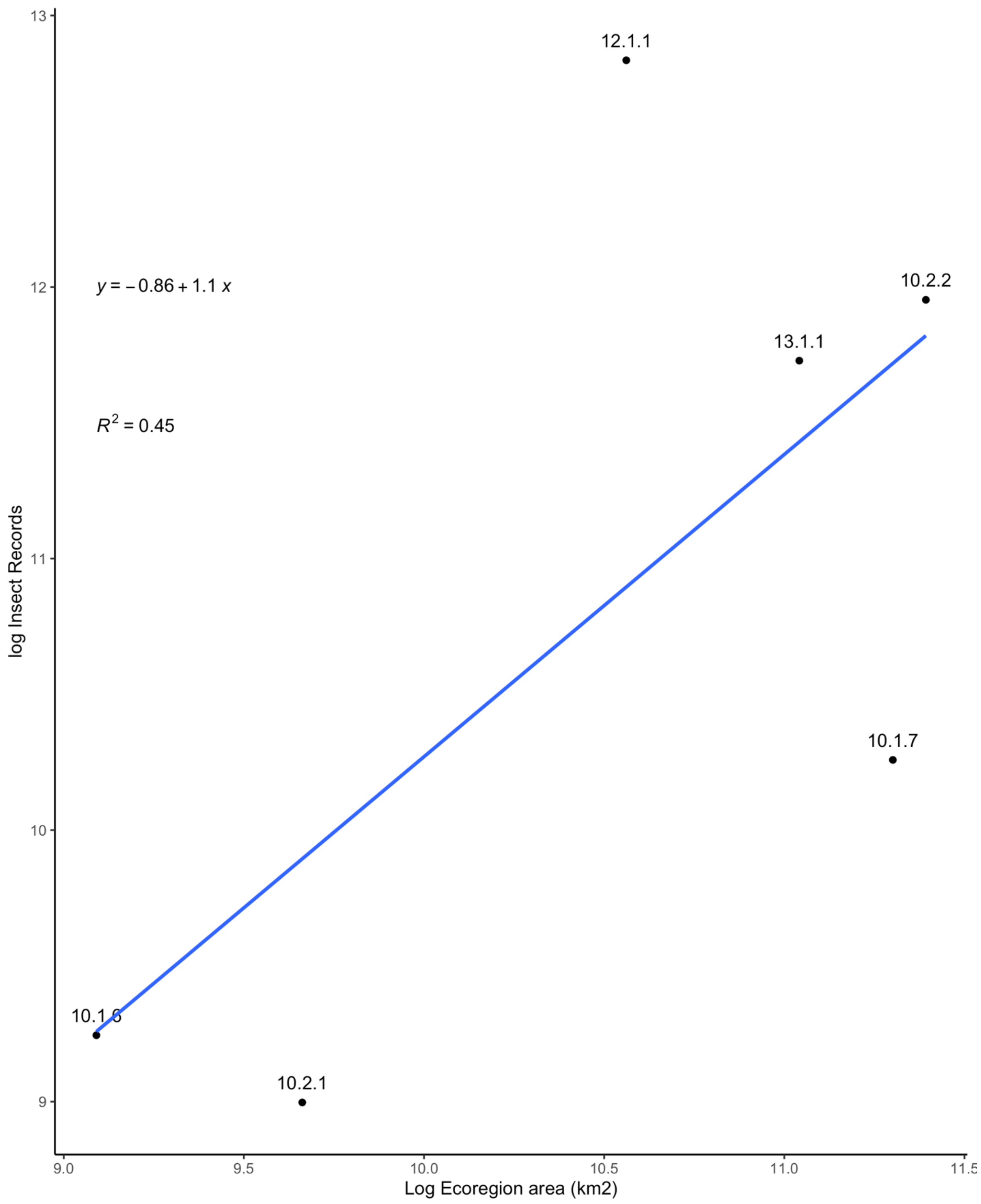

12.1.1
10.2.2
13.1.1
10.1.7
10.1.6
10.2.1
$y = -0.86 + 1.1\,x$
$R^2 = 0.45$
log Insect Records
Log Ecoregion area (km2)
13
12
11
10
9
9.0
9.5
10.0
10.5
11.0
11.5
